# Unlocking the power of antimicrobial peptides: advances in production, optimization, and therapeutics

**DOI:** 10.3389/fcimb.2025.1528583

**Published:** 2025-04-28

**Authors:** Mohd Sadeeq, Yu Li, Chaozhi Wang, Feifei Hou, Jia Zuo, Peng Xiong

**Affiliations:** Biosynthesis and Bio Transformation Center, School of Life Sciences and Medicine, Shandong University of Technology (SDUT), Zibo, China

**Keywords:** antimicrobial peptide, AMPs, antibiotic resistance, SPPS, SUMO, Pichia pastoris

## Abstract

Antimicrobial peptides (AMPs) are critical effectors of innate immunity, presenting a compelling alternative to conventional antibiotics amidst escalating antimicrobial resistance. Their broad-spectrum efficacy and inherent low resistance development are countered by production challenges, including limited yields and proteolytic degradation, which restrict their clinical translation. While chemical synthesis offers precise structural control, it is often prohibitively expensive and complex for large-scale production. Heterologous expression systems provide a scalable, cost-effective platform, but necessitate optimization. This review comprehensively examines established and emerging AMP production strategies, encompassing fusion protein technologies, molecular engineering approaches, rational peptide design, and post-translational modifications, with an emphasis on maximizing yield, bioactivity, stability, and safety. Furthermore, we underscore the transformative role of artificial intelligence, particularly machine learning algorithms, in accelerating AMP discovery and optimization, thereby propelling their expanded therapeutic application and contributing to the global fight against drug-resistant infections.

## 1 Introduction

**Figure 1:**
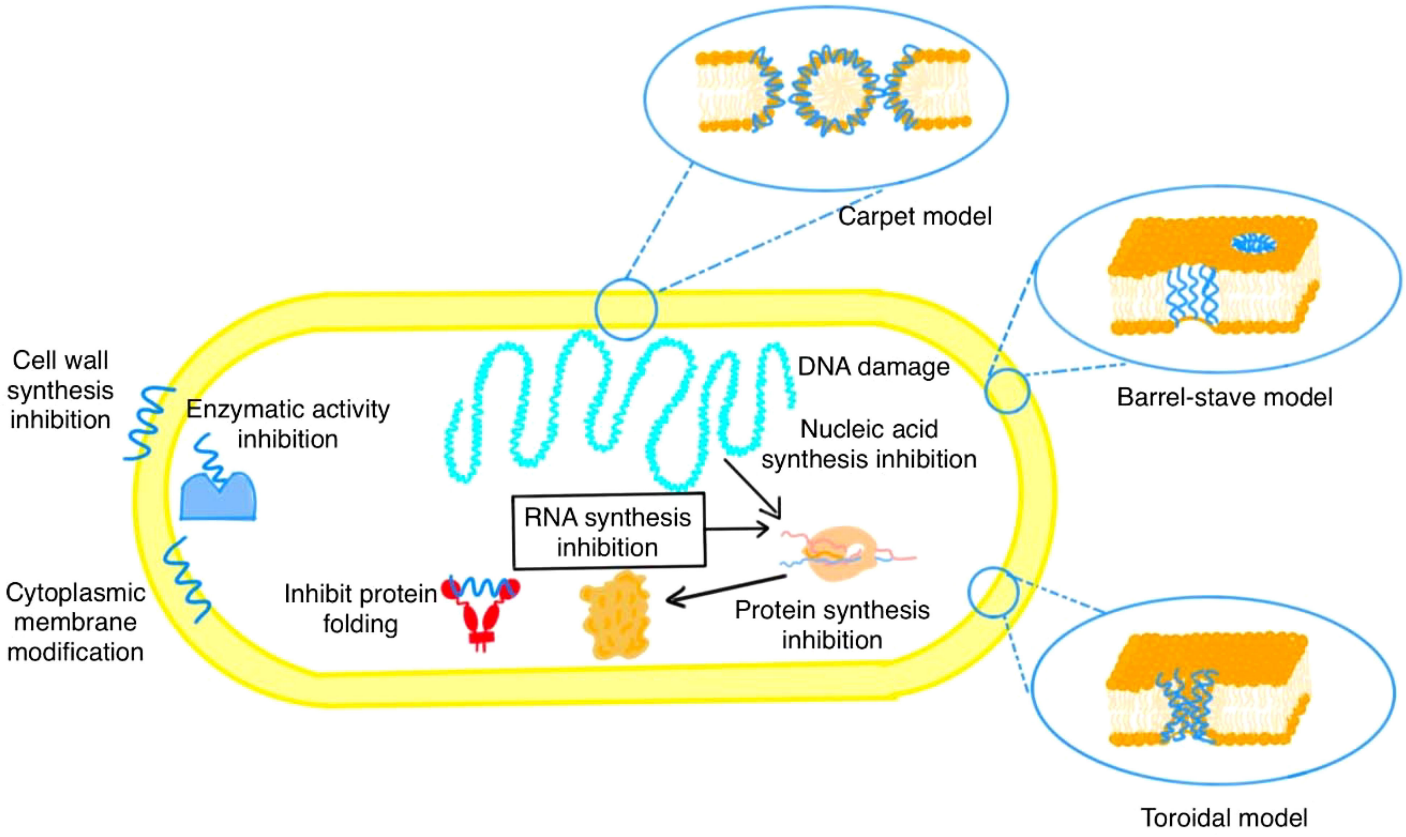
Proposed mechanisms of antimicrobial peptide (AMP) action. AMPs target bacterial cells through various mechanisms, including disruption of the cell membrane and wall. These include (i) membrane pore formation via the Barrel-stave, Toroidal, and Carpet models, (ii) inhibition of cell wall synthesis, and (iii) modification of the cytoplasmic membrane. Intracellularly, AMPs affect (iv) enzymatic activity, (v) protein folding, (vi) protein synthesis, and (vii) nucleic acid/RNA synthesis, as well as inducing microbial DNA damage.

Antibiotic resistance, a rapidly escalating global health crisis, stands as one of the most significant challenges facing global health today, with an estimated 4.9 million deaths attributed to it in 2019 alone (Yang et al., 2018; Haney et al., 2019; Bellavita et al., 2023; Darby et al., 2023; Helmy et al., 2023; Salam et al., 2023; Almutairy, 2024; Viebrock et al., 2024). Bacterial resistance to antibiotics develops through intrinsic mechanisms (drug inactivation, target alteration, and efflux pumps) or is acquired via spontaneous mutations and horizontal gene transfer (Laws et al., 2019; Romo and Quirós, 2019). The emergence of antibiotic-resistant bacterial strains, first observed in 1940 with penicillin-resistant *Staphylococcus aureus* (Foster, 2017) has continuously escalated the global burden of antimicrobial resistance. The 2022 Global Burden of Disease study found that six bacteria— *Escherichia coli* (E. coli), *Staphylococcus aureus* (S. aureus), *Klebsiella pneumoniae* (K. pneumoniae), *Streptococcus pneumoniae* (S. pneumoniae), *Acinetobacter baumannii* (A. baumannii), and *Pseudomonas aeruginosa* (P. aeruginosa) were responsible for 73% of deaths from antimicrobial resistance in 2019, emphasizing the need for urgent medical solutions (Murray et al., 2022; Ho et al., 2024). With antibiotic resistance on the rise, the development of alternative antimicrobial therapies ideally with broad-spectrum efficacy, low toxicity, novel mechanisms, and new targets has become imperative (Murugaiyan et al., 2022; Aparicio-Blanco et al., 2024). Natural AMPs (AMPs) leveraging their multifunctional activity against diverse pathogens and minimal propensity for resistance evolution, exemplify pioneering solutions to transcend the limitations of conventional antibiotics (James et al., 2019; Margit Mahlapuu and Ekblom, 2020; Dwivedi et al., 2024; Purohit et al., 2024). AMPs are small, potent, broad-spectrum antimicrobials found across diverse life forms, from microbes to higher eukaryotes (Thomas and Antony, 2024). AMPs are generally short peptides, typically comprising around 100 amino acid residues with molecular weights under 5,000 Da, although some can be larger, reaching 130-150 residues (Hancock and Diamond, 2000; Zasloff, 2002; Mukesh Pasupuleti and Malmsten, 2012; Viruly et al., 2023). Gramicidin, derived from *Bacillus brevis*, was the first identified AMP (Yang and Yousef, 2018). Since its discovery, numerous other natural peptides with broad-spectrum activity against various pathogens (bacteria, viruses, fungi, and protozoa) have been identified (Datta and Roy, 2021). The Antimicrobial Peptide Database (APD; http://aps.unmc.edu/AP), which includes over 3,000 natural AMPs, serves as a crucial resource for identifying innovative therapeutic candidates (Wang et al., 2022a; Cresti et al., 2024). Similarly, the Data Repository of AMPss (DRAMP; http://dramp.cpu-bioinfor.org/), which provides a significantly larger database of over 30,000 AMP entries, including experimentally validated data on stability, hemolytic activity, and cytotoxicity. Based on their structural characteristics, AMPs, can be categorized into different groups, including linear peptides, cyclic peptides, and those containing disulfide bridge (Koehbach and Craik, 2019; Odunitan et al., 2023). These molecules often adopt distinct secondary and tertiary structures, frequently featuring post-translational modifications that are essential for their biological activity (Xuan et al., 2023; Vincenzi et al., 2024a). Many of these peptides require specific metal ions, such as calcium or magnesium, to maintain their active conformation and for optimal antimicrobial activity (Ngoc et al., 2023; Adriana et al., 2024). These structural and functional adaptations allow AMPs to disrupt microbial membranes through mechanisms such as the barrel-stave (transmembrane pores), toroidal pore (peptide-lipid cooperative pores), and carpet models (surface lysis) (Melo et al., 2009; Cruz et al., 2013) (Li et al., 2021b). As illustrated in **Figure 1**, cationic AMPs initially bind bacterial membranes via electrostatic attraction to anionic phospholipids. At high concentrations, this interaction causes direct membrane destabilization, whereas lower concentrations promote endocytic entry (Cudic and Otvos, 2002). Following internalization, AMPs target cytoplasmic enzymes, DNA, and RNA, disrupting replication and metabolic processes (Cudic and Otvos, 2002) (Steinstraesser et al., 2011; Haney et al., 2017; Divyashree et al., 2020). This multifaceted activity, including immunomodulatory effects, distinguishes them from traditional antibiotics (Torres et al., 2019). These properties have led to the Food and Drug Administration (FDA) approval of several peptide-based therapeutics, some of which are summarized in **Table 1**, while AMPs in various clinical phases are detailed in **Table 2**. Despite their potential, significant challenges remain in translating AMPs from research to clinical application. Despite their therapeutic potential, significant hurdles impede the transition of AMPs from research to clinical application (Marsian and Lomonossoff, 2016; De Oliveira et al., 2023). A primary challenge lies in producing therapeutically viable AMPs at scale (Jia et al., 2017). Large-scale production is essential not only to generate sufficient quantities for clinical use but also to enable thorough characterization of AMP composition, structure, function, and mechanisms of action. Such comprehensive characterization is critical for understanding interactions with target pathogens and optimizing efficacy and safety. Early AMP research relied on isolating these peptides from natural sources like cecropins from silk moth, *Hyalophora cecropia* to assess their activity (Qu et al., 1982; Bulet and Stocklin, 2005) and melatinin from bee venom (*Apis milifera)* (Smith et al., 1988). Extracting AMPs directly from natural sources, while yielding highly active molecules, presents several challenges. These include low, stress-dependent concentrations, slow and costly processing, potential environmental impact, and product impurities (Deng et al., 2017; Mazurkiewicz-Pisarek et al., 2023). In contrast, chemical synthesis offers high yields and purity, but it is expensive, especially for peptides requiring post-translational modifications. Furthermore, synthesizing longer peptides (over 50 residues) is complex and prone to sequence errors due to the stepwise addition of amino acids (Boutin et al., 2019; Wibowo and Zhao, 2019). These limitations highlight the need for cost-effective, efficient methods to produce large quantities of high-purity, therapeutically viable peptides. Heterologous production, involving the cloning of AMP-coding genes into expression vectors and their introduction into host organisms, offers a scalable and cost-effective strategy to meet this demand (Tripathi and Shrivastava, 2019; Lei et al., 2021). However, careful selection of the expression system is crucial, as the choice of host organism can significantly impact yield, cost-effectiveness, and the biological activity of the produced AMP (Ji et al., 2017; Muthunayake et al., 2020a). Wild-type *E. coli*, for example, is commonly used but unsuitable for cysteine-rich AMPs due to its inability to efficiently form disulfide bonds (Kim et al., 2020). Similarly, yeast species like *Pichia pastoris* are being explored for their ability to perform post-translational modifications essential for proteins and extracellular secretion (Garcia-Ortega et al., 2020). Another challenge in AMP development lies in their inherent instability and susceptibility to degradation (Luong et al., 2020). Optimizing these peptides is inherently complex, as improvements to one property (e.g., antimicrobial potency) often come at the expense of others (e.g., safety or stability). For example, structural attributes necessary for bacterial membrane disruption can inadvertently increase toxicity toward host cells, limiting therapeutic utility (Sarah et al., 2005; Kryczka and Boncela, 2018; Wu et al., 2024).

**Table 1:** FDA-Approved Antimicrobial Peptides (AMPs), Their classes, target pathogens, and therapeutic uses.

S. No	Antimicrobial Peptide (AMP)	Class	Target Pathogens	Therapeutic Use	Approval Year	References
1	Gramicidin D	Linear peptide antibiotic	Gram-positive bacteria	Topical treatment for infected wounds	1955s	(Hallett et al., 1956; Usmani et al., 2017; Lee et al., 2018; Chen and Lu, 2020)
2	Polymyxin B	Cyclic lipopeptide	Gram-negative bacteria (e.g., *Pseudomonas aeruginosa*)	Treatment for multi-drug-resistant infections (topical, ocular)	1950s	(Falagas and Kasiakou, 2005; Yu et al., 2021; Buchholz et al., 2024)
3	Daptomycin	Cyclic lipopeptide	Gram-positive bacteria (e.g., *Staphylococcus aureus*, *Streptococcus*)	Treatment of complicated skin infections and bacteraemia	2003	(Kelesidis, 2014; Heidary et al., 2017; Frankenfeld et al., 2018; Faure et al., 2024)
4	Colistin (Polymyxin E)	Cyclic lipopeptide	Gram-negative bacteria (e.g., *Acinetobacter*, *Klebsiella*, *Pseudomonas*)	Last-resort treatment for multi-drug-resistant Gram-negative infections	1959, reintroduced later	(El-Sayed Ahmed et al., 2020; Yang et al., 2024)
5	Bacitracin	Cyclic polypeptide	Gram-positive bacteria (e.g., *Streptococcus*, *Staphylococcus*)	Topical treatment for skin infections	1948	(Oleru et al., 2023)
6	Nisin	Bacteriocin (antibiotic)	Gram-positive bacteria, some Gram-negative species	Preservative in food products (GRAS status)	1969	(Shin et al., 2016; Field et al., 2021; Yuan et al., 2024)
7	Melittin (in development)	Peptide from bee venom	Broad-spectrum (including bacteria, fungi, and viruses)	Antimicrobial, anticancer, immunomodulatory (in trials)	N/A	(Saini et al., 1999; Zhang et al., 2024)
8	Surotomycin	Lipo- polypeptide cyclic	Treatment of *Clostridium difficile* infections	Membrane disruption of Gram-positive bacteria	Advanced Phase III trials (FDA decision pending)	(Alam et al., 2015; Boix et al., 2017; Aziz et al., 2019)
9	Dalbavancin	Lipoglycopeptide (semi synthetic)	Treatment of acute bacterial skin and skin structure infections caused by Gram-positive bacteria, including *MRSA*	Inhibits cell wall synthesis by binding to D-Ala-D-Ala terminus	2014	(Black et al., 2023)
10	Plazomicin	Semisynthetic, amino glycoside	Treatment of complicated urinary tract infections (CUTI), including those caused by multidrug-resistant bacteria	Aminoglycoside that binds to bacterial ribosomes and inhibits protein synthesis	2018	(Carter et al., 2000)
11	Eravacycline	Synthetic flourocycline	Treatment of complicated intra-abdominal infections	Tetracycline derivative that inhibits bacterial protein synthesis	2018	(Lee and Burton, 2019)

**Table 2:** Overview of antimicrobial peptides in clinical development: mechanisms, applications, and current trial status.

Antimicrobial Peptide (MP)	Administration Route	Mechanism of Action	Clinical Application	Clinical Application	Company
Human lactoferrin-derived peptide hLF1-11	Intravenous	Binds to DNA	Conditions linked to LPS-mediated diseases and fungal infections	Phase 1 (completed)	AM-Pharma
Histatin-1 and -3, P-113 (Histatin derivatives)	Topical, Mouthwash	Generates reactive oxygen species	Chronic P. aeruginosa infections, gingivitis, and periodontal diseases	Phase 1, Phase 2–3	Demgen
LTX-109 (Lytixar)	Topical	Disrupts bacterial membrane	Uncomplicated skin infections by Gram-positive bacteria, MRSA and MSSA nasal carriage	Phase 2	Lytix Biopharma
Opebacan (rBPI21, Neuprex)	Intravenous	Disrupts bacterial membrane	Burns, wounds, and meningococcal infections	Phase 2 (completed)	Xoma
Opebacan (rBPI21, Neuprex)	Intravenous	Disrupts bacterial membrane	Post-traumatic infections	Phase 2 (failed)	Xoma
Omiganan (MBI-226, MX-594AN)	Topical	Disrupts bacterial membrane	Catheter-associated infections	Phase 3 (completed)	Migenix
Omiganan (MBI-226, MX-594AN)	Topical	Anti-inflammatory	Rosacea, severe acne, vulval epithelial neoplasia, genital warts	Phase 2b (completed)	Migenix, Biofrontera
EA-230	Topical	Immunomodulatory	Sepsis, endotoxemia	Phase 2 (recruiting)	Exponential Biotherapies
IMX942 (Dusquetide)	Topical	Immunomodulatory	Oral mucositis	Phase 3 (recruiting)	Inimex Pharmaceuticals
PMX-30063 (Brilacidin)	Topical or Intravenous	Disrupts bacterial membrane	Acute bacterial skin infections	Phase 2	Innovation Pharmaceuticals
OP-145	Topical (Ear Drops)	Neutralizes bacterial toxins	Chronic otitis media	Phase 2 (completed)	OctoPlus
XF-73	Topical	Disrupts bacterial membrane	Staphylococcal infections in surgeries, nasal carriage	Phase 2 (recruiting)	Destiny Pharma
XOMA-629	Topical (Gel)	Disrupts bacterial membrane	Impetigo	Phase 2a	Xoma
DPK 060	Topical	Not Available (NA)	Infections in eczematous lesions	Phase 2 (completed)	DermaGen AB
Murepavadin (POL7080)	Intravenous	Targets outer membrane LPS transport protein D	Lower respiratory infections by P. aeruginosa, ventilator-associated pneumonia	Phase 3 (recruiting)	Polyphor
Surotomycin	Oral	Depolarizes bacterial membrane	C. difficile-associated diarrhoea	Phase 3 (completed)	Cubist Pharmaceuticals, Merck & Co.
Iseganan (IB-367)	Aerosol, Mouthwash	Disrupts bacterial membrane	Ventilator-associated pneumonia, oral mucositis	Phase 3 (failed)	Intrabiotics Pharmaceuticals
XMP 629	Topical	Not Available (NA)	Acne	Phase 3 (failed)	Xoma
Talactoferrin (TLF, rhLF)	Oral	Not Available (NA)	Sepsis	Phase 3 (suspended)	Agennix
Pexiganan (Locilex)	Topical or Intravenous	Disrupts bacterial membrane, stimulates defensin production	Infections by diabetic foot ulcers	Phase 3 (failed)	Ganaera
P2TA	Intravenous	Immunomodulatory	NA	Phase NA	

Another challenge in AMP development lies in their inherent instability and susceptibility to degradation Another challenge in AMP development is optimization for therapeutic use. Optimizing these peptides is inherently complex, as improvements to one property (e.g., antimicrobial potency) often come at the expense of others (e.g., safety or stability) (Luong et al., 2020; Wang et al., 2022b). For instance, hydrophobicity and amphipathicity, crucial for disrupting bacterial membranes, can also increase cytotoxicity (Boto et al., 2018; Wu et al., 2024). Similarly, while cost-effective linear l-peptides are susceptible to proteolysis, more stable, modified peptides are expensive to produce (Silva et al., 2011; Chow et al., 2019). Strategies such as cyclization, incorporation of unnatural amino acids, and terminal modifications have been employed to address these limitations. Numerous reviews have explored various facets of AMPs, including sequence, structure, activity, selectivity, toxicity, mechanisms of target and host cell interaction, and optimization strategies (Silva et al., 2011; Torres et al., 2019; Ting et al., 2020a; Mitchell et al., 2021). This review provides a comprehensive overview of established AMP production methods, including chemical synthesis and recombinant heterologous expression, aiming for high yield and purity. We also summarize key AMP design and optimization strategies crucial for enhancing the therapeutic effectiveness of these promising molecules.

## 2 Antimicrobial peptide synthesis

Initial AMP isolation involved low-yield extraction from substantial quantities of natural biological materials (Brand et al., 2013). Large-scale AMP production is now feasible through chemical synthesis or recombinant expression. While recombinant methods are generally preferred for longer peptides (>50 residues) due to cost-effectiveness (Guzmán et al., 2007), chemical synthesis remains essential for peptides requiring specific modifications or exhibiting toxicity in recombinant systems (often necessitating fusion proteins) (Ma et al., 2023). Although chemical synthesis allows precise sequence control, heterologous production is a more scalable approach for obtaining larger quantities of modified peptides. The advantages and challenges of each method are analyzed below.

### 2.1 Chemical synthesis of peptides

**Figure 2:**
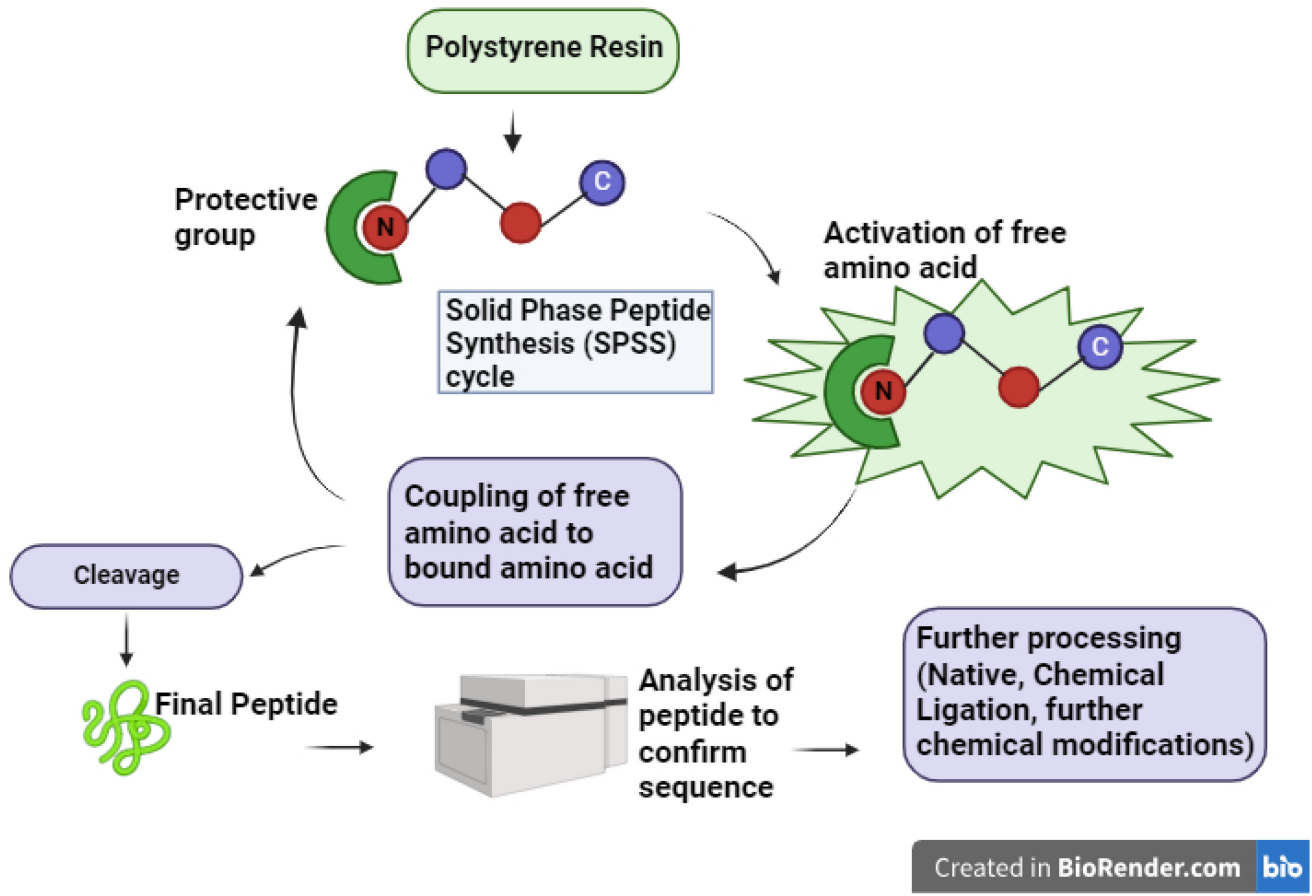
Overview of the Solid Phase Peptide Synthesis (SPPS) cycle. This schematic illustrates the essential steps in SPPS, starting with the activation of free amino acids and their sequential coupling to resin-bound amino acids. Following peptide elongation, the peptide is cleaved from the resin, analyzed to verify the sequence, and subjected to further processing, such as native chemical ligation or additional chemical modifications, to obtain the final, desired peptide product.

**Figure 3:**
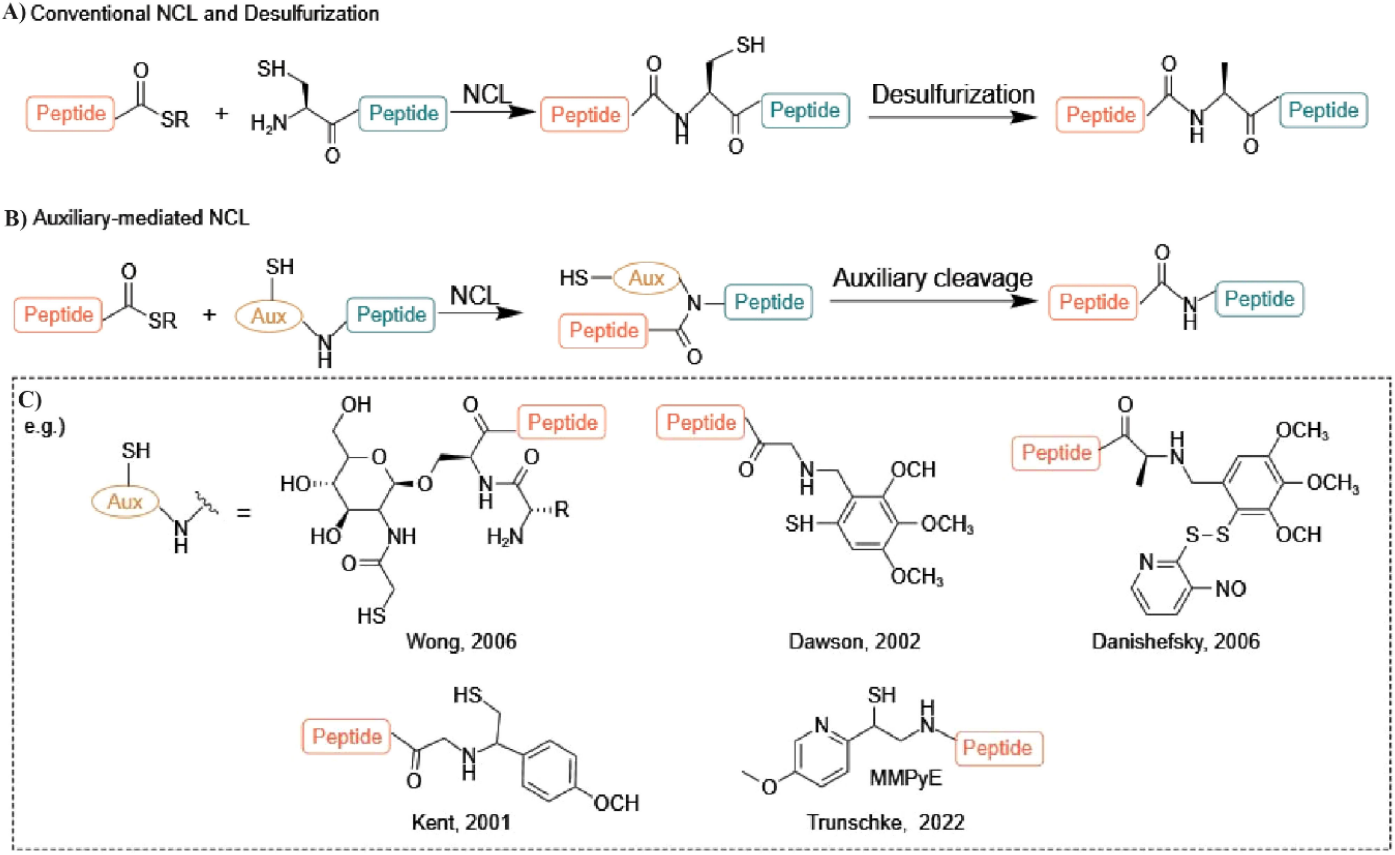
**(A)** Schematic representation of native chemical ligation (NCL) and desulfurization, illustrating native peptide bond formation through trans-thioesterification and S-to-N acyl shift mechanisms. **(B)** Auxiliary-mediated NCL, facilitating ligation followed by cleavage for peptide synthesis. **(C)** Examples of common auxiliaries introduced by Wong [1], Kent [2], Danishefsky [3], and MPyE auxiliary, developed by Trunschke et al. [4], enabling NCL at sterically hindered junctions and expanding the scope of peptide synthesis.

Chemical AMP synthesis offers advantages over natural extraction by enabling precise sequence modification, including incorporation of non-natural amino acids, for enhanced activity and stability studies (Haney et al., 2017). Peptides can be chemically synthesized via solution-phase or solid-phase methods (Kim and Mcalpine, 2013; Ollivier et al., 2017). Solid-phase peptide synthesis (SPPS) is often preferred for modified peptides due to simplified production and purification (Jaradat, 2018; Boutin et al., 2019). In SPPS, the growing peptide chain is anchored to a solid support (e.g., polystyrene resin) via a cleavable linker. Iterative deprotection and coupling reactions extend the chain, with wash steps removing excess reagents and by-products. Final cleavage from the resin yields the purified target peptide (**Figure 2**) (Coin et al., 2007; Lacroix and Li-Chan, 2014). Efficient peptide synthesis is crucial for high-throughput cell-based assays. SPOT synthesis, an automated and cost-effective method (López-Pérez et al., 2017), addresses this by directly generating peptides on a porous membrane, enabling both production and protein-protein interaction screening. This *in situ* synthesis involves sequential addition of protected amino acids, followed by deprotection and cleavage (Coin et al., 2007; Nandhini et al., 2022). Fmoc (9-fluorenylmethyloxycarbonyl) and Boc (tert-butoxycarbony) are the two most popular choices for protecting the N (α) group during this process (Noki et al., 2024). While both are effective, the Fmoc strategy is often preferred due to its milder deprotection conditions and the ability to simultaneously cleave both the linker and side chain protecting groups (Behrendt et al., 2016; Giesler et al., 2020). Synthesizing longer peptides, particularly AMPss exceeding 50 amino acids, remains challenging despite advancements in SPPS synthesis. Native chemical ligation provides an alternative by joining peptide fragments through a thioester-cysteine reaction, forming a native peptide bond (Yan and Dawson, 2001; Wan and Danishefsky, 2007; Bondalapati et al., 2016; Cistrone et al., 2019; Tian et al., 2022). This bond formation proceeds via trans thio esterification followed by an S-to-N acyl shift. Native chemical ligation (NCL), while useful, requires N-terminal cysteine, a residue with low natural abundance (∼1.7% in human proteins) and often unsuitable distribution for ligation. **Figure 3** illustrates the principles of native chemical ligation (NCL) and desulfurization, along with examples of commonly used auxiliaries. Traditionally, NCL proceeds linearly from C- to N-terminus, developments such as desulfurization and auxiliary-mediated ligation overcome these limitations (**Figure 3A**) (Macmillan, 2006; Zuo et al., 2015; Agouridas et al., 2019; Sun and Brik, 2019; Kent, 2021). The Kent group pioneered the use of chemical auxiliaries, or non-native thiol modifications, for peptide ligation at Xaa-Gly and Gly-Xaa junctions. Their method employed a removable N-terminal oxyalkyl moiety, proceeding through a six-membered ring intermediate, and cleaved using zinc (Canne et al., 1996). Drawing upon Dawson’s auxiliary-mediated ligation strategy, Danishefsky’s group devised a cysteine-free native chemical ligation technique applicable to the synthesis of both N- and O-linked glycopeptides (Offer et al., 2002; Wu et al., 2006). Aimoto and colleagues introduced a photolabile auxiliary that is compatible with and cleavable under mild conditions for polypeptide synthesis (Kawakami and Aimoto, 2003). The Wong group introduced sugar-assisted ligation as a specific application of auxiliary-mediated ligation (Côté et al., 2007). A thio-sugar auxiliary, incorporated as a glycosyl amino acid during solid-phase peptide synthesis, facilitates amide bond formation near the ligation site via thiol exchange with a peptidyl thioester. Subsequent desulfurization removes the auxiliary (Yan and Dawson, 2001). Successfully synthesized via a two-step native chemical ligation/desulfurization approach, the AMPs murepavadin displayed potent activity against *P. aeruginosa* clinical isolates (Chaudhuri et al., 2021). An auxiliary developed by Seitz et al. facilitates ligation at sterically hindered junctions, enabling the synthesis of antimicrobial proteins such as DCD-1L and opistoporin-2. This SPPS-compatible auxiliary is cleavable under mild basic conditions (Loibl et al., 2015). The utility of these advancements has been demonstrated in the synthesis of a 120-amino acid peptide containing eight MUC5AC tandem repeats, achieved via the ligation of two 60-residue segments (Trunschke et al., 2022). These developments highlight the continuous evolution of auxiliary-mediated NCL, expanding its applicability for the synthesis of structurally complex peptides and proteins. These and other studies clearly demonstrate that the auxiliary-mediated approach effectively extends native chemical ligation, enabling the synthesis of complex, functionalized proteins with enhanced structural and functional diversity.

Expressed protein ligation is a strategy that combines recombinant protein production with native chemical ligation to overcome NCL’s size limitations. This involves ligating a recombinant protein thioester and a synthetic peptide with an *N*-terminal cysteine (Muir et al., 1998). Native chemical ligation has enabled successful synthesis of complex peptides and proteins, including siglec-7 (Izumi et al., 2014), human interleukin-6 glycoprotein (Reif et al., 2014), and anti-microbial peptide caenopore-5 (Medini et al., 2015). For a more comprehensive understanding of these synthetic approaches, readers are encouraged to refer to the relevant literature (Chalker, 2013; Fernández-Tejada et al., 2015; Raibaut et al., 2015; Bondalapati et al., 2016; Thompson and Muir, 2020; Wang and Cole, 2020). This approach is particularly useful in chemical and molecular biology for preparing proteins with defined post-translational modifications, such as glycosylation.

### 2.2 Heterologous expression system and strategies to upscale the AMP production

**Figure 4:**
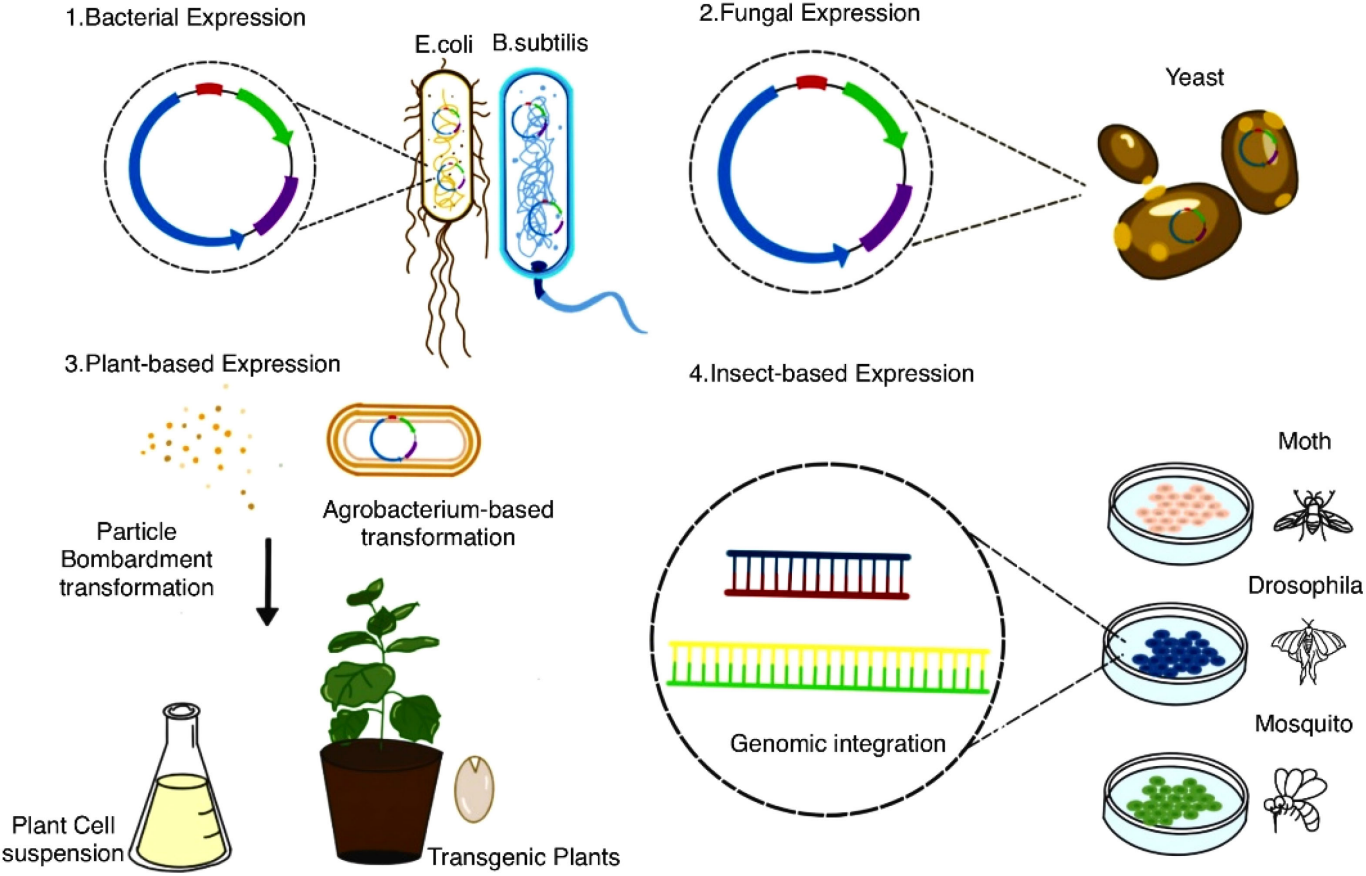
Expression systems for recombinant protein production. This diagram illustrates various expression systems used for recombinant protein production. (1) *Bacterial expression* utilizes bacteria, while *Yeast expression* relies on yeast cells for protein production. (2) *Plant expression* encompasses techniques like *Agrobacterium gene transformation* and *particle bombardment transformation*, enabling the creation of transgenic plants or plant cell suspensions. (3) *Insect expression* involves common insect hosts for genomic integration and protein expression. These systems represent diverse approaches for producing recombinant proteins in different biological hosts.

Heterologous expression involves introducing AMP genes into non-native hosts—such as bacteria, yeast, plant, or insect cells—to leverage their rapid growth and synthesis machinery (**Figure 4**). This approach has transformed these organisms into versatile biofactories, enabling scalable AMP synthesis (Hood, 1997). Below, we explore key heterologous systems (**Table 3**), highlighting their unique advantages and applications for AMP production.

**Table 3:** Comparison of antimicrobial peptide production systems: natural hosts vs. recombinant methods.

S. No	Advantage	Disadvantage	Reference
**Natural Host**	- Triggered by immune response - Non-toxic to cells - Activated through proteolytic cleavage - Specific to certain tissues	- Low production yields	(Bals et al., 1998; Frick et al., 2014; Wan et al., 2016; Blotnick et al., 2017)
**Solid-phase Synthesis**	- No protease activity during synthesis - Often results in higher or equal activity - High purity achievable	- Expensive process - Some peptides are difficult to synthesize	(Pedersen et al., 2012; Behrendt et al., 2016)
**Bacterial Production**	- High production rates - Cost-effective - Well-established method	- Susceptible to protease degradation - Risk of endotoxin contamination - Lacks post-translational modifications	(Simonen and Palva, 1993; Inoue et al., 2007; Magalhaes et al., 2007; Li et al., 2009; Yi et al., 2015)
**Yeast Production**	- Efficient secretion system - Supports post-translational modifications - High expression yields - Economical - Suitable for fermentation processes	- Risk of excessive glycosylation	(Hong et al., 2007; Çelik and Çalık, 2012; Vogl and Glieder, 2013; Meng et al., 2017b)
**Plant Production**	- Potential for large-scale production - Low-cost production - Capable of post-translational modifications - Can use cell suspension cultures	- Difficult to genetically modify - Long cultivation periods - Limited yields and stability	(Hood, 1997; Morassutti et al., 2002; Sun et al., 2011; Engler et al., 2014)
**Insect Cell Production**	- Can be expressed from genomic or plasmid sources - Enables post-translational modifications	- Low expression yields - Expensive process - Scalability challenges - Potential issues related to the lytic cycle	(Jarvis, 2003; Hu et al., 2005; Zitzmann et al., 2015)

Comparative Overview of Antimicrobial Peptide Production Methods: Advantages and Disadvantages of Natural Hosts, Solid-phase Synthesis, and Recombinant Expression Systems.

#### 2.2.1 Bacterial expression system

Bacteria are popular hosts for heterologous expression due to their rapid growth, cost-effectiveness, and efficient production (Zerbs et al., 2009; Deng et al., 2017; Burdette et al., 2018). However, some bacterial systems face limitations in secreting AMPs, forming disulfide bonds, and performing complex modifications like glycosylation (Zhang et al., 2019; Pratama et al., 2021). Various bacteria, including *Corynebacterium* (Salim et al., 1997), *Streptomyces* (Xu and Zhang, 2014; Myronovskyi and Luzhetskyy, 2019), and *Pseudomonas* (Loeschcke and Thies, 2015) can serve as hosts for heterologous expression. These hosts, in conjunction with different promoters and vectors, offer a range of options for peptide production. This section focuses on the most common bacterial hosts: *E. coli, Bacillus subtilis*.

*Escherichia coli* is a bacterial workhorse, widely employed for efficiently producing recombinant peptides, proteins, and membrane proteins (Parachin et al., 2012a; Wibowo and Zhao, 2019). However, producing complex molecules, especially small, positively charged peptides, pushes *E. coli* to its limits. These peptides are vulnerable to degradation, contamination, and misfolding due to the bacterium’s limited post-translational modification capabilities (Terpe, 2006; Chen, 2012; Kaur et al., 2018). To overcome these challenges, selecting an appropriate *E. coli* strain is crucial (Hayat et al., 2018). Genetically engineered strains, (**Table 4**) optimized for protein production, offer advantages like enhanced mRNA stability, cytoplasmic disulfide bond formation, reduced protease activity, and compatibility with challenging gene expression. These advantages can significantly improve peptide expression (Baneyx, 1999; Berkmen, 2012). For example, a study comparing the production of two recombinant defensins (HD5 and LAP) in *E. coli* strains *BL21* and *Origami B* found that *HD5* production was significantly higher in *BL21*, with superior quality and stability. This highlights how strain selection can profoundly impact recombinant protein production (López-Cano et al., 2022).

**Table 4:** Commonly used engineered *E. coli* strains for heterologous protein production.

Strain	Source	Features	References
BL21	Derived from B834	Capable of high-yield protein production with straightforward induction protocols.	(Doherty et al., 1995)
BL21	Evolved from BL21	Contains a mutation in rne131 gene to stabilize mRNA, enhancing protein expression.	(Makino et al., 2011)
BL21-pLys	Based on BL21	Ideal for expressing toxic proteins and displays resistance to chloramphenicol.	(Schwarz et al., 2004)
Origami	Derived from K-12	Mutations in trxB and gor genes allow for enhanced disulfide bond formation inside the cytoplasm.	(Makino et al., 2011)
Origami B	From K-12	Includes trxB, gor, and lacZY mutations to further improve protein production.	(Kaplan et al., 2016)
Rosetta	Derived from BL21	Facilitates expression of eukaryotic genes containing codons rarely used in E. coli.	(Fathi-Roudsari et al., 2016)
Rosetta-gami	Based on BL21	Supports both eukaryotic protein expression and enhanced disulfide bond formation for better protein folding.	(Parker et al., 2020)
C41	Natural mutant of BL21	A natural variant designed for efficient overexpression of membrane-bound and toxic proteins.	(Dumon-Seignovert et al., 2004; Schlegel et al., 2021)
C43	Natural mutant of BL21	Another variant adapted for high-level expression of membrane-bound and toxic proteins.	(Dumon-Seignovert et al., 2004; Tian et al., 2009a)
Rosetta 2-pLys	Evolved from BL21	Boosts eukaryotic protein expression by using plasmid-based rare codons and supports toxic protein expression.	(Kopanic and Schal, 1999)
Shuffle	From E. coli B (C3029) & E. coli K-12 (C3026)	Assists in proper folding of proteins with disulfide bonds directly in the cytoplasm.	(Lobstein et al., 2012)
Tuner	BL21-based	Lacks lactose permease (LacY), allowing for tighter control of protein expression levels.	(Binder et al., 2016)
Lemo21	Derived from BL21	Specifically designed for problematic proteins like membrane and toxic proteins, improving their solubility.	(Schlegel et al., 2012)
SoluBL21	Based on BL21	Optimized for expressing soluble mammalian proteins, ensuring proper folding.	(Hata et al., 2013)
W3110	K-12 variant	Common wild-type strain frequently used in laboratory settings.	(Khankal et al., 2008)
HMS174	K-12-based	Contains a recA mutation, which enhances plasmid stability during protein expression.	(Bell and Kowalczykowski, 2016)
BLR	Modified from BL21(DE3)	A recA-deficient version that prevents unwanted recombination, improving plasmid stability.	(Goffin and Dehottay, 2017)

Producing AMPs in bacteria like *E. coli* often involves fusing the AMP to a carrier protein (Li et al., 2007; Pavelka et al., 2024). This strategy has proven particularly useful for AMPs that exhibit high toxicity to bacterial hosts, such as challenging ones like the potent cathelicidin LL-37 (Pavelka et al., 2024), protegrin-1 (Azari et al., 2020a) and the modified bovine lactoferricin known as LfcinB-W10 (Feng et al., 2010), by shielding them from premature breakdown within the bacterial cell.

To optimize recombinant expression in *E. coli*, careful selection of both the bacterial strain and expression vector is essential, guided by a thorough understanding of the target peptides characteristics. This includes considering the desired level of basal expression determined by the vector’s promoter (Lozano Terol et al., 2021). The choice of expression vector significantly impacts recombinant protein production in *E. coli*. Key considerations include the promoter system, which directly influences expression levels. Popular choices include the lac, tac, and lacUV5 promoters (utilized in vectors like pGEX, pMAL, and pUC), as well as the powerful T7 promoter found in the pET system (Rosano and Ceccarelli, 2014; Tucci et al., 2016; Du et al., 2021). Additionally, some vectors incorporate fusion tags to simplify purification or detection. Careful selection of these elements is crucial for optimizing yield and functionality (Zhang et al., 2022).

*Bacillus subtilis (B. subtilis)*, a Gram-positive, endotoxin-free bacterium, offers a safer alternative to *E. coli* as a recombinant protein expression system (Schallmey et al., 2004). Its well-characterized protein secretion machinery makes it particularly suitable for the production and secretion of AMPs. Researchers explored the potential of *B. subtilis* and its efficient secretion pathways for producing recombinant AMPs and AMP fusion proteins extracellularly (Harwood and Cranenburgh, 2008; Taguchi et al., 2015; Rosales-Mendoza et al., 2016; Yang et al., 2016; Neef et al., 2021) (Fu et al., 2018; Zhang et al., 2018). A recent study reported the successful production of the cecropin A-melittin mutant in *B. subtilis*, with a yield of 159 mg/l of purified CAM-W (Ji et al., 2017). Two separate studies highlight the successful production of AMPs in *Bacillus subtilis* WB800N. One study (Zhang et al., 2015) produced the fungal defensin plectasin by fusing its gene with SUMO, 6xHis, and *sacB* signal peptide sequences within the *pGJ148* shuttle plasmid. This method yielded 41 mg/L of fusion protein, which upon purification and SUMO protease digestion, resulted in 5.5 mg/L of 94% pure, biologically active plectasin. Another study successfully expressed and secreted PR-FO, a different AMPs, achieving yields of 16 mg/L and 23 mg/L of fusion protein using SP*amyQ* and SP*sacB* signal peptides, respectively (Zhang et al., 2020). *Bacillus subtilis* presents a promising system for industrial applications due to its beneficial characteristics. Recent publications have explored various approaches to enhance and optimize its use in these contexts (Westers et al., 2004; Kovács, 2019; Su et al., 2020; Lei et al., 2021; Put et al., 2024).

#### 2.2.2 Yeast based expression system

Yeast offers a robust eukaryotic platform for AMPs (AMP) production, combining advantages absent in bacterial systems. Unlike prokaryotes, yeast integrates efficient secretion systems that minimize toxicity by exporting AMP extracellularly, while enabling essential post-translational modifications (PTMs) like glycosylation to enhance peptide stability and bioactivity (Vieira Gomes et al., 2018a; Kumar and Kumar, 2019a; Zhao et al., 2024).

Producing AMPs in non-native hosts presents several challenges. AMP production poses challenges due to their toxicity to expression systems (e.g., *E. coli*), instability, protease susceptibility, and post-translational modification requirements (Ingham and Moore, 2007; Da Cunha et al., 2017). While *E. coli* can be engineered for AMP production, the process can be costly and time-consuming, leading researchers to explore alternative expression systems (Schreiber et al., 2017). Yeast presents a compelling alternative, combining beneficial aspects of both eukaryotic and prokaryotic systems. These advantages include efficient protein secretion, post-translational modifications, and rapid growth (Mattanovich et al., 2012b; Parachin et al., 2012a; Schreiber et al., 2017; Vieira Gomes et al., 2018a; Lee et al., 2008; Kwak, 2024). *Saccharomyces cerevisiae*, first employed for recombinant protein production in the 1980s, remains a widely utilized host due to its well-characterized genetics, physiology, and fermentation processes, along with its GRAS status (Mattanovich et al., 2012b; Kastberg et al., 2022a). *Pichia pastoris* and *Saccharomyces cerevisiae* are the two most widely adopted yeasts for recombinant protein production, offering advantages such as adaptability to large-scale fermentation and the ability to produce biologically active eukaryotic proteins (Tran et al., 2017). The *Saccharomyces cerevisiae* expression system has been successfully employed for the production of various therapeutic proteins, including vaccines against hepatitis B and hantavirus, as well as insulin and other human hormones (Tomoko et al., 1985; Hahm and Chung, 2001; Antoniukas et al., 2006; Dimiceli et al., 2006; Baeshen et al., 2014). Traditional yeast expression systems, while widely used, present limitations such as fermentative metabolism, hyper glycosylation, and potentially low protein yields (Wagner and Alper, 2016; Wang et al., 2017; Baghban et al., 2019). The limitations of traditional yeast expression systems have led to the development of alternative systems utilizing unconventional yeasts. Among these, *Pichia pastoris* (*P. pastoris*) has emerged as a preferred host for heterologous protein expression (Zhu et al., 2019). This is reflected in the dramatic increase in studies utilizing *P. pastoris* in recent years (Bill, 2014; Wagner and Alper, 2016; Baghban et al., 2019). Unlike *S. cerevisiae*, which diverts significant carbon towards ethanol production, *P. pastoris* efficiently uses carbon for growth, leading to high biomass, and channels its resources towards recombinant protein production (Vieira Gomes et al., 2018a; Walker and Pretorius, 2018; Karbalaei et al., 2020a; Parapouli et al., 2020; Barone et al., 2023; Popova et al., 2023). Proteolytic degradation of recombinant proteins in *K. phaffii (P. pastoris)* typically occurs during vesicular transport or after secretion into the extracellular space, leading to reduced yields and compromised activity of the target protein (Werten et al., 2019; Raschmanová et al., 2021). To address protease-related challenges in *K. phaffii*, protease-deficient strains like *SMD1163, SMD1165, and SMD1168* have been developed. These strains exhibit significantly reduced or eliminated protease activity due to targeted gene deletions. However, this advantage comes at the cost of reduced growth rates, lower transformation efficiencies, and decreased viability compared to wild-type strains (Daly and Hearn, 2005; Karbalaei et al., 2020a). Both constitutive and inducible promoters are employed for heterologous gene expression in yeast. However, inducible promoters are generally preferred due to their ability to fine-tune expression levels, leading to higher product yields (Kastberg et al., 2022a). Despite its popularity, offers a limited selection of expression vectors compared to other yeast or bacterial systems. Commonly used vectors for AMP production rely on the methanol-inducible AOX1 promoter, necessitating a two-step process: initial high cell density growth followed by methanol-induced protein expression. However, high yields are achievable with this system, as demonstrated by NZ2114 production reaching 2390 mg/L (Zhang et al., 2014). **Table 5** summarizes various AMPs produced using *Pichia* pastoris as a heterologous host.

**Table 5:** Production of antimicrobial peptides in pichia pastoris and *Saccharomyces cerevisiae*: yield, activity, and expression systems.

Peptide Name	Source	Expression Host	Expression System	Antimicrobial Activity	Yield	Reference
**ABP-dHCCecropin A**	Cecropin, *Hyphantria cunea*	*P. pastoris*	Inducible pPICZαA vector, regulated by AOX1 promoter	Antimicrobial activity	21 mg	(Sang et al., 2017)
**Ac-AMP2**	*Amaranthus caudatus*	*P. pastoris*	Inducible pPICZαA vector	Antifungal activity	210 mg	(Huang et al., 2021)
**Enterocin L50A & L50B**	Enterocin L50 peptides, *Enterococcus faecium*	*S. erevisiae*	Inducible pYABD01 vector, controlled by PGAL 1 promoter	Activity against Gram-positive bacteria	0.023 mg	(Basanta et al., 2010)
**MiAMP1**	*Macadamia integrifolia*	*P. pastoris*	Inducible pPICZαA vector	Antifungal activity	210 mg	(Huang et al., 2021)
**MP1106, designed**	Defensin from *Pseudoplectania nigrella*	*P. pastoris*	Inducible pPICZαA vector, AOX1 promoter	Antimicrobial activity	8.31 mg	(Cao et al., 2015)
**Mytichitin A**	From *Mytilus coruscus*	*P. pastoris*	Inducible pPICZαA vector, modified AOX1 promoter	Activity against Gram-positive bacteria	18.2 mg	(Meng et al., 2016)
**Neutral protease 1**	From *Oryza sativa*	*P. pastoris*	Inducible pHBM905BDM vector, modified AOX1 promoter	Antibacterial activity	Mg	(Ke et al., 2012)
**PaDef**	From Mexican avocado	*P. pastoris*	Inducible pPICZαA vector, AOX1 promoter	Activity against Gram-positive and Gram-negative bacteria	79 mg	(Meng et al., 2017a)
**Snakin-1**	Plant antimicrobial peptide	*P. pastoris*	Inducible pPIC9 vector, AOX1 promoter	Broad-spectrum antibacterial activity	40 mg	(Kuddus et al., 2016)
**Tilapia piscidin 4**	*Oreochromis niloticus*	*P. pastoris*	Inducible pPICZαA vector, AOX1 promoter	Antimicrobial and immunomodulatory activity	0.002 mg	(Tai et al., 2021)
**LL-37**	Human cathelicidin peptide	*P. pastoris*	Inducible pPICZαA vector, AOX1 promoter	Broad-spectrum antimicrobial activity	28.63 mg/L	(Zhan et al., 2021a)
**LIG**	Synthetic antimicrobial peptide	*P. pastoris*	Inducible pPICZαA vector, AOX1 promoter	Broad-spectrum antimicrobial activity	5.9 mg/L	(Zhao et al., 2024)
**Plectasin**	From *Pseudoplectania nigrella*	*P. pastoris*	Inducible pPICZαA vector, AOX1 promoter	Antibacterial activity	426.3 mg/L	(Liang et al., 2022)
**Hepcidin**	Human peptide	*P. pastoris*	Inducible pPICZαA vector, AOX1 promoter	Antimicrobial and iron regulation	ND	(Qiao et al., 2023).

Microbial systems, while advantageous for biopharmaceutical production, face challenges due to high-mannose N-glycosylation (Amann et al., 2019; Kulagina et al., 2021). This modification can impact protein half-life and potentially trigger immune responses (Chung et al., 2022). Glycoengineering is often needed to optimize the therapeutic potential of *P. pastoris*-produced proteins. Genetically engineered yeasts can now perform human-like N-glycosylation, including terminal sialic acid addition (Wildt and Gerngross, 2005; De Pourcq et al., 2010; De Wachter et al., 2021). The engineered yeast strain YSH44, devoid of endogenous glycosylation machinery, was employed to establish a humanized glycosylation pathway. This involved introducing genes encoding key enzymes for human N-glycosylation, including mannosidases I and II, N-acetylglucosaminyltransferases I and II, and a UDP-GlcNAc transporter. This engineered pathway enables the production of proteins bearing the human-like oligosaccharide GlcNAc2Man3GlcNAc2 (Logez et al., 2012; Felber et al., 2014).

#### 2.2.3 Plants

**Figure 5:**
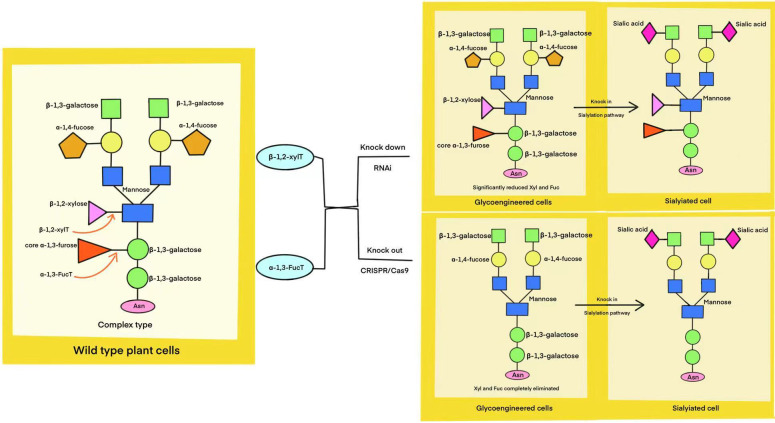
Glycoengineering strategies for humanizing plant N-glycans. This diagram illustrates the process of modifying wild-type plant cells to generate human-like N-glycan structures. By knocking down (RNAi) or knocking out (CRISPR/Cas9) specific genes, glycosylation pathways are altered to reduce undesired plant-specific modifications. Further genetic engineering, such as the introduction of the human sialylation pathway, allows for the incorporation of sialic acids, resulting in fully humanized N-glycans.

Plants are emerging as a promising platform for producing various biomolecules, including AMPs. Producing recombinant AMPs in plants aims to achieve high AMP concentrations and enhance disease resistance in transgenic plants (Abranches et al., 2005; Nawrot et al., 2014; Zou et al., 2023; Chekan et al., 2024). Plants possess the cellular machinery necessary for essential post-translational modifications, including glycosylation, disulfide bond formation, and proteolytic cleavage (Webster and Thomas, 2012; Tschofen et al., 2016; Margolin et al., 2020). These modifications are crucial for the activity of various proteins, including AMPs (Pouresmaeil and Azizi-Dargahlou, 2023). Additionally, the presence of chaperones similar to those found in animals ensures proper protein folding, further supporting their suitability as a production platform (Shinmyo and Kato, 2010; Da Cunha et al., 2014; Pouresmaeil and Azizi-Dargahlou, 2023). Plant-based heterologous protein production, while advantageous, faces challenges due to differences in glycosylation patterns compared to mammalian cells (Gomord et al., 2010; Karki et al., 2021b). Plant-specific N-glycans, characterized by the presence of mannose, α-1,3-fucose, and β-1,2-xylose, can trigger immunogenic responses in humans (Strasser, 2016; Srisangsung et al., 2024). This poses a potential concern for the use of plant-derived therapeutics, as pre-existing antibodies against these glycans in human serum could lead to adverse immune reactions. Therefore, mitigating plant-specific glycosylation is crucial for the successful development of plant-based platforms for therapeutic protein production, particularly for heterologous AMP production (Shanmugaraj et al., 2021a; Dubey et al., 2023). To address the challenges of plant-specific glycosylation, researchers are developing glyco-engineered plant cell lines. These engineered lines, often generated using RNAi or CRISPR/Cas9 technology, lack the enzymes α-1,3-FucT and β-1,2-XylT, enabling the production of humanized proteins (Mercx et al., 2017; Karki et al., 2021a) as shown in **Figure 5**. AMP production in plants can be achieved through stable or transient transgene expression in whole plants or plant cell suspensions, utilizing either plastid or nuclear transformation (Hoelscher et al., 2022; Raimondo et al., 2023; Chaudhary et al., 2024). Stable transformation integrates foreign genes into the plant genome (nuclear or plastid), enabling heritable trait transfer (Bélanger et al., 2024). Jin et al. engineered two different plant species, *Arabidopsis thaliana* and *Medicago sativa*, to produce recombinant β-gallinacin-3, a cysteine-rich AMPs (Jin et al., 2018). In *M. sativa*, transgenic plants were generated after codon optimization and vector construction. Subsequent analysis revealed that the minimum inhibitory concentrations against three bacterial strains were 32, 16, and 128 μg/mL, respectively (Jin et al., 2022). This highlights the potential of plant systems for producing functional AMPs. Similar strategies, such as expressing a CBD-dermasptin B-1 fusion peptide in tobacco hairy roots, have also demonstrated enhanced antimicrobial activity against phytopathogens, particularly *Alternaria alternata*, compared to the unmodified peptide (Shams et al., 2019). These findings underscore the potential of plant-based expression systems for improving the efficacy of AMPs against specific targets. While advantageous for cost-effective peptide production due to seed-based accumulation, nuclear transformation in plants necessitates transgene containment strategies to mitigate the risk of outcrossing with native species (Horn et al., 2004). Chloroplast-based expression, or transplastomic expression, offers a promising alternative for producing peptides (Hoelscher et al., 2022). Molina et al. successfully produced high levels of a viral antigenic peptide in tobacco chloroplasts, demonstrating this approach’s potential for vaccine development (Molina et al., 2004). Transplastomic expression offers stable transgene inheritance, high protein yields, and multigenic expression capabilities. While lacking glycosylation pathways, chloroplasts facilitate proper protein folding and disulfide bond formation, enabling functional peptide production (Egelkrout et al., 2012; Abiri et al., 2016; Moustafa et al., 2016). Maternal inheritance further reduces transgene escape. Although stable transformation was once a standard method for biomolecule production, its lengthy timeline, particularly for establishing transgenic plants, prompted the search for faster alternatives. This led to the emergence of transient heterologous expression as a more efficient method for producing recombinant peptides (Marsian and Lomonossoff, 2016; Liu et al., 2018). Transient expression offers a faster and more flexible approach to producing heterologous peptides, partly because it avoids the complexities of long-term gene integration and expression (Egelkrout et al., 2012; Clark and Maselko, 2020). Established plant cell transformation protocols, particularly biolistic, contribute significantly to the widespread adoption of transient expression (Anami et al., 2013). This method employs DNA-coated microprojectiles to effectively deliver genetic material into plant cells, facilitating the generation of transgenic plants across a wide range of species, including challenging monocotyledons (Su et al., 2023). Agroinfiltration presents an effective alternative for delivering recombinant vectors into plant cells. This technique utilizes *Agrobacterium tumefaciens*, a bacterium with a unique ability to transfer genes into a host plant’s genome (Gelvin, 2017). For example, researchers were able to introduce a gene encoding the antifungal peptide Rs-AFP2 from radish (*Raphanus sativus)* into rice (*Oryza sativa L*. cv. Pusa basmati 1). This successful transfer holds promising implications for improving fungal disease resistance in rice (Jha and Chattoo, 2010). Plant-based AMP production has been achieved using various parts, including the successful expression of human lactoferrin (hLF) in tobacco hairy roots (Shanmugaraj et al., 2021a). Carrot suspension cell cultures served as the platform for producing taliglucerase-α, marking a milestone, as this first plant-derived pharmaceutical protein production method eliminates the need for further processing to modify glycosylation patterns after protein synthesis. The efficacy of taliglucerase alfa has been clinically proven (Manniello et al., 2021). A number of authoritative articles on strategies for heterologous expression of AMPs in plants have been reviewed elsewhere (Stączek et al., 2023).

#### 2.2.4 Insect cells

Insect-derived AMPs, offer a promising solution to bacterial resistance. Insects produce a diverse array of AMPs, including attacins, lebocins, and moricins, with the first identified AMP, cecropin, discovered in insects in 1980. The baculovirus expression system, utilizing insect cells, has a long and established history in producing a variety of heterologous proteins, including AMPs (Berger and Poterszman, 2015; Van Oers et al., 2015; Santry et al., 2017). Since Smith’s ground breaking 1983 study showcasing the use of *Autographa californica* multiple nucleopolyhedrovirus for heterologous protein production (Smith et al., 1983), the baculovirus expression system has evolved into a powerful protein production tool. This progress is driven by improvements in recombinant virus isolation, cell culture methods, and glycoprotein modification strategies (Kost et al., 2005; Joshi et al., 2021) (Kost et al., 2005; Joshi et al., 2021). Recombinant baculoviruses are proving useful beyond the production of important bioactive products, emerging as versatile tools for gene delivery and gene therapy applications (Wang et al., 2024). While insect cells offer several advantages for recombinant production, including efficient post-translational modifications and folding While insect cells offer several advantages for recombinant production, including efficient post-translational modifications and folding (Fabrick and Hull, 2017), their glycosylation capabilities are limited (Contreras-Gómez et al., 2014). Notably, they lack the enzymes required for terminal galactose and sialic acid addition (Palomares et al., 2021), which can impact the function of some recombinant proteins (Betenbaugh et al., 1996; Fernandes et al., 2021). Insect cell lines like Sf9 and Tni, while capable of N-glycosylation, produce simpler paucimannosidic structures due to the trimming activity of their *FDL*-encoded *β-N*-acetylglucosamines. To overcome this limitation, various attempts have been made. One approach involves introducing mammalian genes into insect cells, equipping them to build more complex glycans. For example, the engineered *Sfβ4GalT* cell line produces a human glycosyltransferase, enabling the addition of galactose to proteins (Jarvis, 2003; Chavez-Pena and Kamen, 2018). Another attempt focuses on disabling the gene responsible for the trimming enzyme. Silencing the *fdl* gene in *BmN4-SID1* cells via RNA interference is one such approach, which has led to the production of glycoproteins with more complex N-glycan structures (Chavez-Pena and Kamen, 2018). Ongoing efforts aim to engineer insect cells to produce therapeutic peptides with improved glycosylation for enhanced effectiveness.

The CRISPR-Cas9 system was successfully employed to edit the *fdl* gene in Sf9 and BmN insect cells. This involved expressing Cas9 with the IE1 promoter and introducing specific guide RNAs (sgRNAs) with the DmU6:96Ab and BmU6-2 promoters (Mabashi-Asazuma and Jarvis, 2017b). An engineered insect cell line, based on Sf9 cells, expresses five mammalian glycosyltransferases. This allows the cells to produce recombinant proteins with complex, terminally sialylated N-glycans, resulting in mammalian-like glycosylation and higher overall glycosylation levels compared to proteins produced by the original Sf9 or High Five cells (Legardinier et al., 2005; Meng et al., 2016). Baculovirus-insect cell systems offer an effective alternative for producing AMPs, particularly those toxics to bacteria or fungi. This approach bypasses limitations of prokaryotic expression systems. For example, it has successfully produced human β-defensin- (Valore et al., 1998), the N-terminal portion of bovine lactoferrin (Valore et al., 1998) and snakin-1 (Zitzmann et al., 2017) and osmotin, a pathogenesis-related plant protein (Zitzmann et al., 2017). While producing the AMPs gloverin from the wax moth in microbial systems has proven challenging due to its inherent antibacterial activity, a study successfully achieved high yields using *Drosophila* cells. This suggests that insect cells may be a more suitable platform for producing insect-derived AMPs (Ikemura, 1985). Recently, scalable batch and perfusion processes were developed for producing BR033, a cecropin-like AMPs from *Lucilia sericata*, using Sf-9 insect cells (Käßer et al., 2022). While insect cell systems offer advantages like post-translational modifications, but their cost-effectiveness compared to established microbial platforms for large-scale production needs further consideration (Plotkin and Kudla, 2011). **Table 6** summarizes various AMPs expressed in insects.

**Table 6:** Production of antimicrobial peptides in insect cell lines: expression systems, application, and yield.

AMP	Source/Origin	Insect Cell Line	Expression System	Vector/Promoter	Application	Yield (mg/L)	Reference
**BRO33**	Lucilia sericata, Cecropin-like AMP	Spodoptera frugiperda (sf9)	Stable, Constitutive	OpIE2 promoter	Antibacterial (against *E. coli*)	Not reported	(Kasse et al., 2022).
**Gloverin**	Galleria mellonella (Lepidopteran)	Schneider 2 cell line	Constitutive	pCoBlast, pMTflGmGlv-V5/His	Antibacterial and Antifungal	25 mg	(Zitzmann et al., 2015).
**Lactoferrin**	Bovine Glycoprotein	Spodoptera frugiperda (sf9)	Constitutive	pGEM-T, pVL1392	Antibacterial	10 mg	(Nakamura et al., 2001).
**Scorpine**	Androctonus australis, Defensin-cecropin-like	Anopheles gambiae (Sua 5.1)	Constitutive	Serpin promoter, pMinHygeGFP	Antibacterial, Antiplasmodial	Not reported	(Carballar-Lejarazú et al., 2020).
**Snakin-1**	Solanum tuberosum	Spodoptera frugiperda (sf9)	Constitutive	pFastBac HT B	Antimicrobial	6.3	(Almasia et al., 2017).

Selected examples of antimicrobial peptides (AMPs) recombinantly produced in insect cell systems, highlighting expression method, activity, and yield.

## 3 Advancements in AMPs production, using heterologous systems

**Figure 6:**
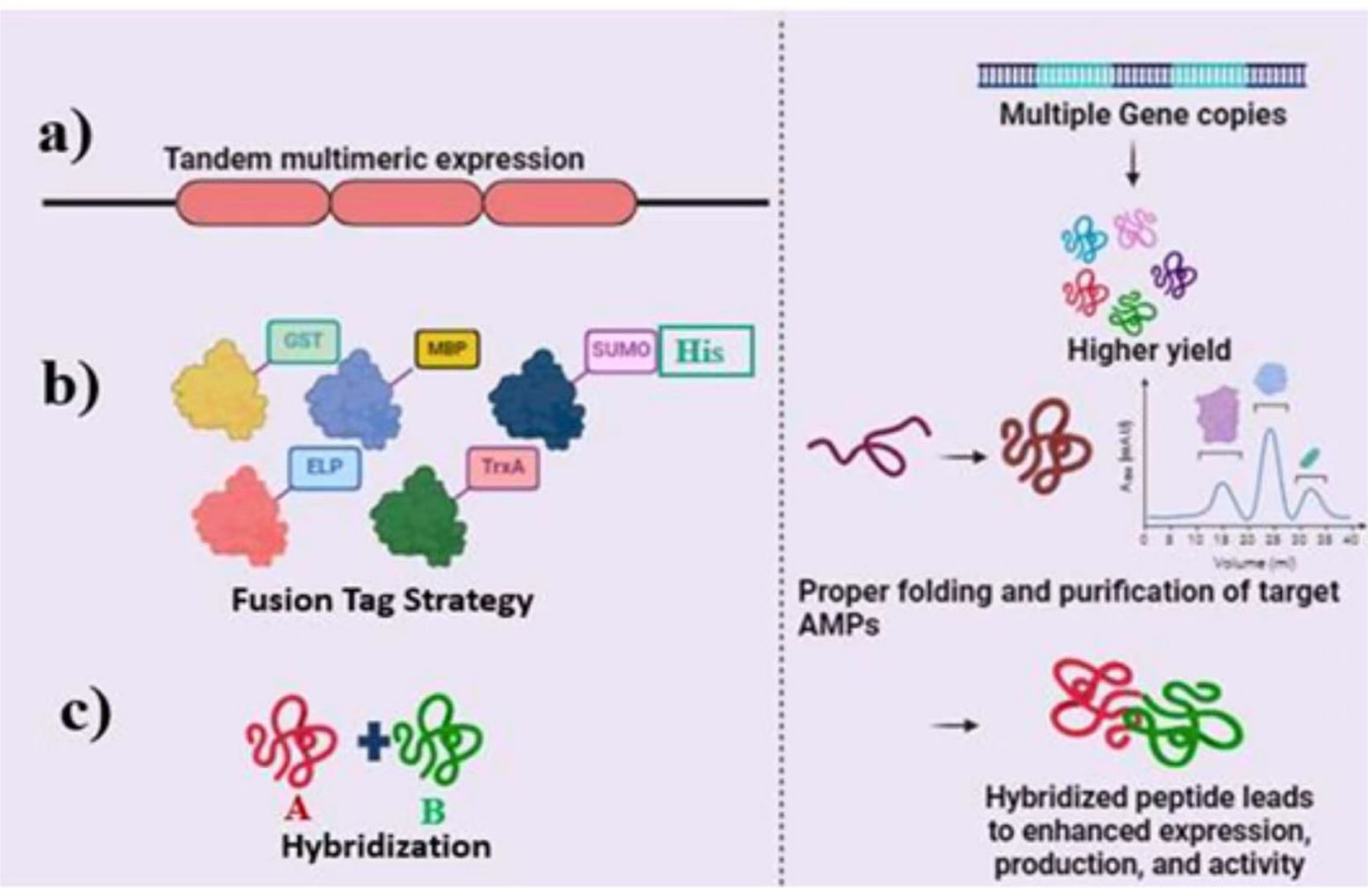
Advanced strategies for optimizing the expression and production of antimicrobial peptides (AMPs). **(a)** *Tandem multimeric expression* leverages multiple gene copies to significantly boost AMP yield. **(b)** *Fusion tag strategy* employs various tags (e.g., GST, MBP, SUMO, His) to enhance the solubility, purification, and correct folding of AMPs, ensuring higher production efficiency. **(c)** *Peptide hybridization* combines distinct peptide fragments to form hybrid AMPs, improving expression levels, biological activity, and overall peptide functionality. These strategies collectively enhance the yield, folding, and purification of active, high-quality AMPs.

The ability to generate substantial quantities of pure AMPs through heterologous expression has revolutionized research in this field, offering a potent combination of cost-effectiveness, streamlined production, and straightforward purification. **Figure 6** illustrates the key strategies for enhancing AMP expression (e.g., tandem multimeric expression, fusion tags, hybridization), while **Table 7** summarizes representative AMPs produced via these methods, their yields, and purification efficiencies. Below, we explore these strategies and their implications for future advancements.

**Table 7:** Expression systems and methodologies for antimicrobial peptides: yields, outcomes, and host-specific strategies.

AMP	Host	Methodology	Outcome	Citations
LL-37	*Pichia pastoris*	Multimeric expression with SUMO fusion tags	154.8 mg/L	(Zhan et al., 2021a)
Cecropin D	*Escherichia coli*	Multimeric expression SUMO fusion	300 mg/L	(Guo et al., 2012; Zhang et al., 2016)
Parasin I-human lysozyme (rehLY-PI)	*Pichia pastoris*	Codon optimization 6×His tag	86 mg/L	(Zhao et al., 2015)
LL37 + Indolicidin	*Pichia pastoris*	6×His-3×FLAG tag	5 mg/L	(Zhao et al., 2024)
Plantaricin EF	hybrid *Pichia pastoris*	6×His tag	32.65 mg/L	(Li et al., 2020a)
CLP (CM4, LL37, TP5)	*Pichia pastoris*	6×His-TEV cleavage	101.71 mg/L	(Castellanos et al., 2020)
NZ2114	*Komagataella phaffii*	AOX1 promoter, high-density culture	2390 mg/L	(Zhang et al., 2014)
Plectasin-Plectasin	*Multimer Escherichia coli*	TrxA fusion	95.1 mg/L	(Cao et al., 2015)
LL-37	*Escherichia coli*	GST/TrxA fusion, codon optimization	0.3 mg/L, 2.4 mg	(Moon et al., 2006; Li, 2013)
LL-37		SUMO/Trxa		
Plectasin	*Bacillus subtilis*	SUMO fusion, SPamyQ/sacB signals	5.5–23 mg/L	(Zhang et al., 2015)
Cecropin A-melittin mutant (CAM-W)	*Bacillus subtilis*	sacB EDDIE fusion	159.46 mg/L	(Ji et al., 2017)
BR033	*Spodoptera frugiperda*	Melittin secretion signal, His_6_ tag fusion protein	Scalable batch production1.5–2.5 mg/L	(Käßer et al., 2022)
Plantaricin 423 (PlaX)	*Saccharomyces cerevisiae*	MFα1 secretion signal, codon optimization	18.4 mg/L; MIC: 1.18 µM (vs. L. monocytogenes)	(Rossouw et al., 2024)
Mundticin ST4SA (MunX)	*Saccharomyces cerevisiae*	MFα1 secretion signal, codon optimization	20.9 mg/L; MIC: 108.52 nM (vs. L. monocytogenes)	(Rossouw et al., 2024)
β-Gallinacin-3	*Medicago sativa (alfalfa)*	Codon optimization, nuclear transformation	MIC: 16–128 µg/mL (vs. bacterial strains)	(Jin et al., 2018)
CBD-Dermaseptin B1	*Nicotiana tabacum (tobacco)*	Fusion with cellulose-binding domain (CBD) for enhanced stability	Improved antifungal activity against Alternaria alternata	(Shams et al., 2019)
Taliglucerase alfa	*Nicotiana tabacum (tobacco)*	Chloroplast expression, glycoengineering	Clinically approved therapeutic protein; no further glycosylation required	(Grabowski et al., 2014)
Rs-AFP2	*Oryza sativa (rice)*	Agroinfiltration with Agrobacterium tumefaciens	Enhanced resistance to Magnaporthe oryzae (rice blast fungus)	(Aerts et al., 2007)

### 3.1 Optimization of codon usage

The genetic code, while nearly universal, exhibits a fascinating quirk called “codon usage bias”. This means that even though multiple codons can code for the same amino acid (due to flexibility at the third nucleotide, or wobble position), organisms tend to favor certain codons over others. This preference for specific codons has been observed across all genomes studied (Hershberg and Petrov, 2008; Chaney and Clark, 2015). Codons used more frequently are deemed preferred or optimal, while less favored ones are termed non-optimal or rare. For instance, mammals tend to favor C or G nucleotides at the wobble position, while budding yeast show a preference for A or U, likely reflecting differences in underlying mutational biases between these organisms (Duret, 2000; Quax et al., 2015). Once viewed as insignificant, codon usage bias is now recognized as a vital layer of genetic regulation. The preference for certain synonymous codons, even within the same gene, can significantly impact both protein levels and structure (Gustafsson et al., 2012; Zhou et al., 2016). Crucial genes, like those for ribosomal proteins, often prefer optimal codons across diverse organisms (Paremskaia et al., 2024). The relationship between codon usage and gene expression is increasingly evident, with genome-wide studies revealing a strong association between codon bias and protein levels. This connection is further supported by experimental manipulations, where optimizing codon usage has resulted in marked increases in protein production across diverse organisms (Peng et al., 2012).

Codon optimization makes genes easier for organisms to read by matching their DNA sequence to the organism’s preferred codons. This process is usually done using specialized computer software (Aguirre-López et al., 2017). Although most results are far more modest, it has been shown that codon optimization can boost protein expression by as much as 1000-fold (Kapust et al., 2002). Species like *E. coli* and yeast have distinct codon preferences due to the amount of tRNAs in their respective genome. Peng and his colleagues have demonstrated that employing codon optimization enhanced the expression of human beta-defensin-2 (hbd2) by a factor of nine, which resulted in a final concentration of 130 mg/L, surpassing the expression levels of human beta-defensin-5 & 6 (hbd5 and hbd6) in *E. coli* (Chadani et al., 2010). Moreover, codons encoding arginine have been found to greatly affect the production of heterologous proteins in *E. coli.* As a result, it is commonly suggested to replace the AGG or AGA codon for arginine with CGT in the target gene (Baghbeheshti et al., 2021). Additional stop codons are added in *E. coli* and yeast employing codon optimization for upscaling the production of AMPs. For example, in *E. coli*, the sequence TAATGA is commonly employed as a termination codon rather than merely TAA or TGA. This modification helps prevent the production of insufficient amounts of AMP products that can occur due to incorrect translational termination (Zhou et al., 2009). Researchers achieved the successful production of two beneficial bacteriocins, namely plantaricin 423 and mundticin ST4SA, using yeast (*S. cerevisiae*) (Rezaei et al., 2020). By optimizing the genes of mundticin ST4SA, the yeast demonstrated enhanced efficacy in combating pathogenic microorganisms. Nevertheless, the above-discussed examples help us to understand and explore the routes to efficiently produce and upscale the production of AMPs.

### 3.2 Tandem multimeric expression

One effective strategy to enhance the production of AMPs is through tandem multimeric expression (Thakker et al., 2011; Shen et al., 2012; Öztürk et al., 2017). This method involves introducing multiple copies of the AMP gene into a host cell, leading to the production of multiple AMP units linked together. This approach, unlike expressing single-unit AMPs, can significantly increase the overall yield of the desired AMP (Zhang et al., 2011). This higher gene dosage leads to increased transcription and, consequently, a higher yield of the desired peptide. The success of this strategy is evident in several studies (**Table 8**). For instance, increasing the copy number of the CM4 gene in *E. coli BL21* significantly enhanced its production. By using pET32 with n 1-8 copies of AMP (CM4), the yield reached 68 mg/L, almost four times higher compared to using a single gene copy (Zhou et al., 2009).Similarly, increasing the gene copy number of ABP-dHC-cecropin A in *E. coli* from one to three resulted in a dramatic yield increase from 0 to 300 mg/L (Panteleev et al., 2017). These examples clearly demonstrate the potential of tandem multimeric expression for enhancing AMP production. This strategy also successfully produced a tetramer fusion of S3 and SΔ3 AMPs in *E. coli BL21* for cost-effective large-scale production of AMPs. Using 8 gene fragment copies, *P. pastoris* released 146 mg/L of plectasin, an AMP with antibacterial action against *S. suis* and *S. aureus* (Seo et al., 2012). These studies demonstrate that both transcription and translational levels increased by employing this strategy. High expression levels due to tandem multimeric genes were seen not only in *E. coliand P. pastoris*, but also in *B. subtilis*. Additionally, using a tandem multimeric approach, *B. subtilis* significantly increased Tachyplesin expression levels (Holaskova et al., 2015). Although the tandem multimeric technique is an effective way to obtain high production of AMPs, the appropriate heterologous expression system must be selected for each AMP. It is also important to stress that there is no linear relationship between the number of copies of a gene and the amount of target protein expressed (Xu et al., 2005; Corrales-García et al., 2020). For instance, soluble expression of hBD2 in *E. coli* yielded conflicting results with 1–4 gene copies, cells containing only one or two joined hBD2 genes (instead of the more common four joined hBD2 genes) achieved a higher level of expression using the pGEX-4T-2 construct, whereas cells with fewer hBD2 gene copies showed a higher growth rate, suggesting that abundant AMP expression may strain the host cells (Almaaytah et al., 2018; Brady et al., 2019). For these reasons, optimizing AMP production in a heterologous expression system requires identifying the optimal gene copy number for a given AMP.

**Table 8:** Tandem multimeric antimicrobial peptides (AMPs) produced in *E. coli* and *Pichia pastoris*.

AMP	Size (kda)	Fusion tags	Host	Yield	References
Buforin II	3.0	CNBr	Escherichia coli	107mg/ml	(Lee et al., 2002)
Bin1b	5.21	Thrombin	Escherichia coli	2.4mg/ml	(Sun et al., 2004)
Indolicidin derivative	1.9	CNBr	Escherichia coli	100mg/ml	(Metlitskaia et al., 2004)
hPAB-b	4.5	Hydroxylamine	Escherichia coli	680 ± 12 mg/ml	(Rao et al., 2005)
Indolicidin	1.9	CNBr	Escherichia coli	0.15mg/ml	(Morin et al., 2006)
β-defensin 2	5.1	CNBr	Escherichia coli	760mg/ml	(Zhong et al., 2006)
Lactoferricin	3.0	CNBr	Escherichia coli	60mg/ml	(Kim et al., 2006)
Cecropin–melittin hybrid	2.3	CNBr	Escherichia coli	89mg/ml	(Wang and Cai, 2007)
Lactoferricin derivative	2.0	CNBr	Escherichia coli	10mg/ml	(Tian et al., 2007)
Histonin	3.0	Furin	Escherichia coli	167mg/ml	(Kim et al., 2008)
LFB15–HP hybrid	3.5	Formic Acid	Escherichia coli	11.3mg/ml	(Tian et al., 2009b)
CM4	4.2	Hydroxylamine	Escherichia coli	12mg/ml	(Li et al., 2011)
LL-37	3.8	Multimeric LL-37 expressed with SUMO fusion tags for enhanced solubility and yield	Pichia pastoris	154.8 mg/L	(Zhan et al., 2021a)
Cecropin D		SUMO		300mg/l	(Zhang et al., 2016)
**Plectasin-Plectasin Multimer**	4.399	TrXa	Escherichia coli	95.1 mg/l	(Jones et al., 2019)
Parasin I (PI)-human lysozyme (hLYS)	18	6X -His tag	Pichia pastoris	86 mg/L	(Zhao et al., 2015)
LL37+Indolicidin	4.8	6x-HIS-3X FLAG	Pichia pastoris	5mg/l	(Zhao et al., 2024)
plantaricin E and plantaricin F	5	6x-HIS	Pichia pastoris	32.65 mg/L	(Li et al., 2020b)
CLP (CM4, LL37and TP5)	4.628	6X-HIS-TEV	Pichia pastoris	101.71mg/l	(Cheng et al., 2023)

This table lists tandem multimeric antimicrobial peptides (AMPs) recombinantly produced in Escherichia coli and Pichia pastoris, outlining their molecular sizes, fusion tags used for expression or purification.

### 3.3 Hybridization expression approach

The hybridization expression strategy is a new method for increasing AMP expression and introducing unique characteristics and synergetic effect. The idea of hybridization expression was employed to combine two AMPs that possess complimentary yet unique characteristics. This fusion resulted in the development of a novel AMP that exhibits improved antimicrobial efficacy while minimizing harm to host cells (Feng et al., 2014). The use of cleavable tags, such as those removable by enzymatic or chemical treatment, or even self-cleaving tags like inteins, allows for the retrieval of the original peptide sequences after purification. These restored peptides can then be utilized together or further separated for individual applications. Numerous studies have highlighted the production fusion AMPS. Cecropins, one of the known AMPs composed of 31-39 amino acids, are frequently used in combination with other AMPs because of their ability to kill both Gram-negative and Gram-positive bacteria (Chen et al., 2009). A novel hybrid AMP, LF15-CA8, comprising amino acid sequences from cecropin A and LfcinB, demonstrated enhanced antibacterial activity against *S. aureus* without causing hemolytic activity (Wang, 2008). Cecropin A (1-8)-magainin2 (1-12) (CA-MA) was synthesized in *P. pastoris*, resulting in a higher yield and exhibited antibacterial activity against a diverse range of microorganisms, including yeasts as well as Gram-positive and Gram-negative bacteria. The peptide cecropin AD, composed of cecropin A and cecropin D, was synthesized in *B. subtilis* with a much higher yield of 30.6 mg/L. It exhibited antibacterial activity against *S. aureus* and *E. coli* (Zhan et al., 2021a).

LL37, the only member of the human cathelicidin family, is extensively found in several human organs and physiological fluids (Hoelscher et al., 2022). LL37 is of significant research interest due to its roles in immunological regulation, immune cell attraction, and wound healing (Engelberg and Landau, 2020; Yang et al., 2020; Ridyard and Overhage, 2021). Several studies demonstrated that the helical structural region consisting of 17-29 residues played a crucial role in LL37’s biological functionality (Engelberg and Landau, 2020; Kielkopf et al., 2020). For example, the hybrid peptide LL-37_Renalexin combines LL-37 and Renalexin, resulting in potent, broad-spectrum antimicrobial activity (Narh et al., 2024), while the hybrid peptide LL-37Tα1, derived from LL-37 and Tα1, acts as a potent anti-endotoxin without hemolytic or cytotoxic activity (Ahmad et al., 2019). Thus, the hybridization expression approach shows great promise for upscaling the AMP production and potent antimicrobial activity.

### 3.4 Fusion tag expression

The choice of host organism significantly influences the production yield of AMPs. While insects, plants, yeast, and bacteria are explored for recombinant AMP production, bacterial (Li, 2011a) and yeast systems (Pipiya et al., 2023) typically offer higher yields. However, AMP production in heterologous hosts faces challenges. Their small size, positive charge, and susceptibility to degradation contribute to cytotoxicity and complicate purification processes in heterologous hosts (Teixeira et al., 2020a; Zainal Baharin et al., 2021; Du et al., 2022; Cesaro et al., 2023; Tang et al., 2023). Intracellular expression of AMPs, often lacking signal peptides, necessitates cell lysis for extraction and purification (Muthunayake et al., 2020a). Fusion protein systems present a common strategy to overcome the challenges associated with AMP production (López-Otín and Matrisian, 2007; Parachin et al., 2012a; Deng et al., 2017). This approach has proven particularly advantageous in bacterial expression systems, enhancing both AMP expression levels and facilitating downstream purification (Costa et al., 2014). Carrier proteins, such as GST, TrxA, and SUMO, act as artificial pro-segments, enhancing solubility, aiding in proper folding, and simplifying purification (Wood, 2014). Once the fusion protein is expressed and purified, tags like GFP and 6 × His, incorporated into the AMP sequence, further facilitate detection and purification (**Figure 6b**). For instance, Thioredoxin, a fusion protein derived from *E. coli*, enhances the solubility of expressed proteins and has been successfully employed in the expression of various AMPs, including epinecidin (Jeyarajan et al., 2024), Snakin-2 (Herbel et al., 2015), stomoxyn ZH1 (Elhag et al., 2017), plectasin (Jing et al., 2010) and Human α-defensin 6 (Wang et al., 2010). GST fusion proteins offer a simple purification method using affinity chromatography with immobilized glutathione, allowing for quick isolation from crude lysates (Zhou et al., 2020). Several studies highlight the effectiveness of GST fusion in enhancing AMP expression. For instance, a GST-Cecropin B fusion expressed in *Pichia pastoris* showed improved bioactivity and yield (Skosyrev et al., 2003). Similarly, in *E. coli*, a GST-Melittin fusion achieved a high yield of 3.5 g/L with 90% purity (De Beer and Vivier, 2008). These findings demonstrate that GST fusion not only enhances the expression of recombinant peptides but also simplifies their purification. While GST fusion proteins often result in soluble expression, achieving optimal solubility may require fine-tuning various factors, including the choice of host strain and optimization of temperature and pH (Lavallie et al., 1993, 2000). Elastin-like polypeptides are versatile fusion carriers that facilitate efficient expression and purification of target proteins due to their unique properties and the strategic incorporation of specific cleavage sites (Varanko et al., 2020). Numerous AMPs, often cationic in nature, have been successfully produced using ELPs (Pereira et al., 2021). For example, CM4 and HβD4 were produced in a bacterial system using an ELP-intein strategy, achieving high antimicrobial activity and a final product yield of 1.84 mg/L (Ghidey et al., 2020). While intein-based approaches have shown promise for maximizing yields, as seen with Pa-MAP2 (Malakhov et al., 2004), chemical cleavage has also demonstrated high yields, such as the 90 mg/L achieved for Hep25 (Butt et al., 2005). Notably, fusing AMPs to ELPs in *Nicotiana benthamiana* resulted in high yields and significantly enhanced antimicrobial activity against *Staphylococcus epidermidis* compared to *E. coli* expression systems (Luengo et al., 2018). SUMO has emerged as a highly effective fusion carrier for recombinant protein production, enhancing both folding and solubility (Azari et al., 2020a; Park et al., 2021a). Its unique protease, SUMO protease 1, recognizes the tertiary structure of the SUMO tag, ensuring precise cleavage and yielding target proteins with native N-termini (Butt et al., 2005; Lee et al., 2008). This system has facilitated the successful expression of challenging proteins, including AMPs like PR-39 (Azari et al., 2020a), Cecropin B (Park et al., 2021a), LL37 (Zhan et al., 2021a), and others (Hoelscher et al., 2022). These findings underscore the importance of considering fusion tag properties and protein-tag interactions when selecting optimal tags for AMP synthesis and purification.

### 3.5 Signal peptide-based secretion

Secreting recombinant proteins directly into the growth medium offers significant advantages over intracellular expression. This strategy minimizes aggregation, reduces host cell toxicity, promotes proper protein folding, and simplifies purification (Freudl, 2018; Duan et al., 2019). Signal peptides, short sequences at the beginning of some proteins, act as “zip codes,” directing them to the cellular export machinery. This guidance ensures efficient transport and prevents premature folding (Yu et al., 2022). Highlighting the impact of signal peptides on protein secretion, a recent study successfully expressed a modified alpha toxin, H35L, as a fusion protein with an NSP4 signal peptide (NSP4_ATH35L) in *E. coli*. By modifying the secretion system through domain swapping and altering the dsbA and pelB signal peptides, the researchers achieved a 3.5-fold increase in the yield of secreted ATH35L (Han et al., 2021). Researchers have also harnessed the efficient SPYncM signal peptide in *B. subtilis* to boost protein secretion. By combining this signal peptide with a dual-promoter system (PHpaII–PgsiB) instead of the typical single promoter, they achieved a remarkable yield of 1.74g/L, demonstrating the potential of tandem promoters for substantial production increases (Gattu et al., 2023). Maeno et al. developed an innovative approach for producing apidaecins using *Streptomyces lividans* (Maeno et al., 1993).

By fusing the AMPs to a protease inhibitor with a cleavable linker, they achieved efficient secretion and simplified purification. This method effectively addressed the challenges of degradation and complex purification often associated with recombinant peptide production (Domhan et al., 2019). The α-factor preproleader has proven highly versatile for functional protein expression in yeast. This system has successfully produced a wide range of proteins, including fungal proteins, green fluorescent proteins, vaccines, (e.g., human insulin), and various AMPs (e.g., LL-37) (Kjeldsen et al., 2002; Fitzgerald and Glick, 2014; Zhan et al., 2021a). Moreover, it has been shown that the increase in the positive charge in the N-domain and high hydrophobicity in the H-domains are potent for efficient secretion system. Furthermore, selecting the desired signal peptide for protein production commonly relies on methods like high-throughput screening or single-specific verification (Zhao et al., 2021). Nevertheless, more work needs to be done in this domain to enhance their yield and maximize their antibacterial activity.

## 4 AMP modifications and *in-vivo* effectiveness

While promising antimicrobial agents, AMPs face challenges in therapeutic applications. Their effectiveness is often limited by protease degradation, binding to serum proteins, and susceptibility to physiological conditions. Additionally, high therapeutic doses can lead to cytotoxicity and hemolysis (López-Otín and Matrisian, 2007). To overcome these limitations, researchers are developing novel peptides with enhanced antibacterial properties and reduced side effects. These efforts focus on modifying naturally occurring AMP sequences using strategies like cyclization, nanoparticle formulations, and incorporation of uncommon amino acids. Chemical synthesis via SPPS is crucial for these modifications, enabling precise sequence control, improved stability, and exploration of structure-activity relationships (Al Musaimi et al., 2022).

### 4.1 Incorporation of unusual amino acids

Chemical synthesis of AMPs offers advantages over natural extraction, enabling precise sequence modification via solid-phase peptide synthesis (Andreu et al., 1985; Pazgier et al., 2006; Pandit and Klauda, 2012; Guan et al., 2019). This facilitates modulating antibacterial potency, exploring structure-activity relationships, and incorporating non-natural amino acids for enhanced activity and stability. For instance, ornithine, 2,4-diaminobutyric acid, and 2,3-diaminopropionic acid can replace lysine to adjust amino acid side chain length (Kumar et al., 2018; Ting et al., 2020a). Vogel et al. showed that substituting lysine with diaminopimelic acid in tryptophan-rich peptides enhanced their antimicrobial activity against *E. coli*. This four-fold increase in efficacy is likely attributed to greater membrane permeabilization (Arias et al., 2018). Cationic peptides incorporating unnatural amino acids demonstrate broad-spectrum antimicrobial activity alongside enhanced resistance to proteolytic degradation. These peptides effectively combat a wide range of pathogens, including multidrug-resistant bacteria, while maintaining stability in biological environments (Glibowicka et al., 2022). Petraccone and colleagues developed a series of cationic synthetic peptides incorporating unnatural amino acids, such as 2-naphthyl-L-alanine and S-tert-butylthio-L-cysteine. These modifications resulted in enhanced antimicrobial activity across a broad spectrum of pathogens while also significantly improving proteolytic stability (Oliva et al., 2018).

**Figure 7:**
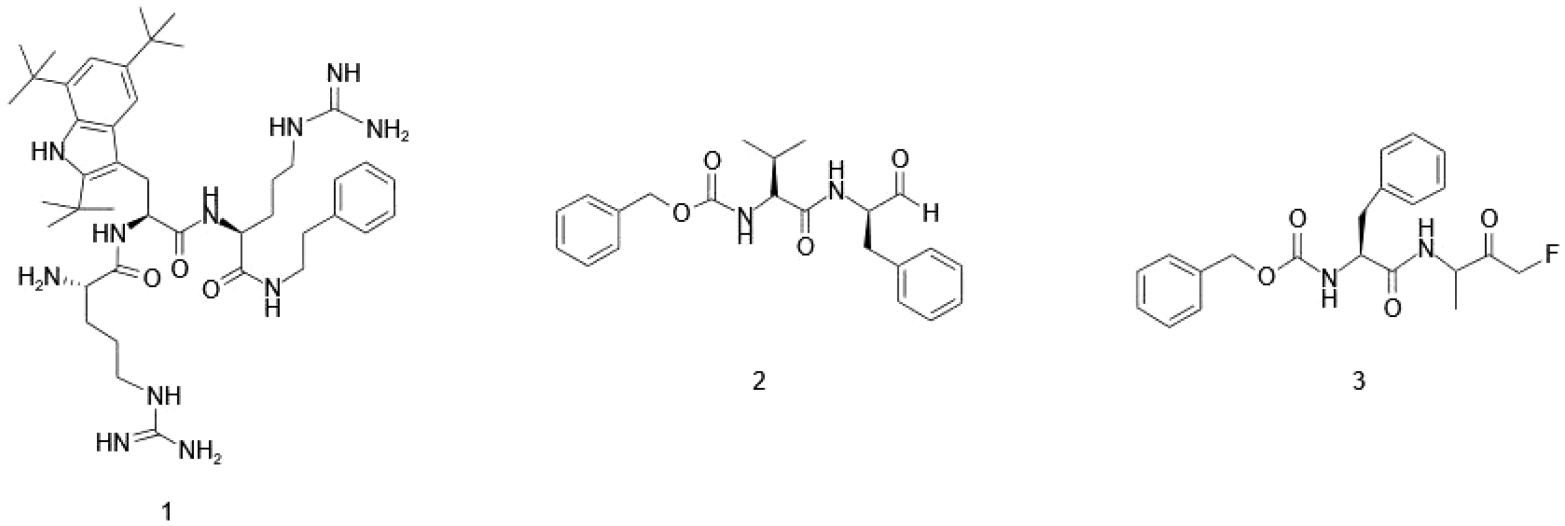
Incorporation of unnatural amino acids for the enhancement of therapeutic peptides. (1) LTX-109: A tri-peptide consisting of a lipophilic, unnatural tryptophan residue flanked by two arginines, illustrating the success of using unnatural amino acids to improve the activity of antimicrobial peptides (AMPs). (2) and (3) Cbz-protected dipeptide calpain inhibitors: Hiromatsu and co-workers repurposed these inhibitors by incorporating unnatural C-terminal amino acids, specifically with fluoromethyl ketone and aldehyde groups, enhancing their inhibitory potency and selectivity.

Building on this approach, LTX-109, a synthetic tripeptide, exemplifies how unnatural amino acid incorporation can optimize antimicrobial efficacy and structural stability (Nilsson Anna et al., 2014). Featuring a non-canonical tryptophan residue flanked by two arginine residues, LTX-109 (**Figure 7** (1)) enhances membrane disruption and protease resistance, reinforcing the potential of synthetic modifications in AMP design. Similarly, dipeptide calpain inhibitors have been modified with unnatural amino acids containing fluoromethyl ketone and aldehyde moieties at the C-terminus of Cbz-protected dipeptides, exhibited notable bacteriostatic activity against *Chlamydia trachomatis*, but this effect was temporary, with bacterial growth resuming after treatment (**Figure 7** (2) and (3)) (Itoh et al., 2019).Together, these studies emphasize the crucial role of unnatural amino acids in designing next-generation AMPs with enhanced potency, stability, and resistance to enzymatic degradation.

Amino acid substitution with proteinogenic L-residues is a common strategy to enhance AMP activity and selectivity. For example, modifying the natural AMP magainin II led to the development of pexiganan, a potent broad-spectrum analogue (Portelinha et al., 2021). This improved activity resulted from substituting specific neutral and anionic amino acids with cationic and hydrophobic residues (Gottler and Ramamoorthy, 2009). Several synthetic AMPs based on L-amino acid substitutions, such as iseganan (protegrin analogue), omiganan (indolicidin analogue), and P113 (histatin analogue), have progressed to late-stage clinical trials.

Substituting L-amino acids with D-amino acids is a promising strategy to enhance the stability and activity of AMPs. Studies have shown that this modification can maintain antimicrobial activity while enhancing protease resistance (Shao et al., 2019; Hasan et al., 2021). For example, a D-enantiomer of the wasp venom AMP, MPI, showed significantly increased protease resistance compared to its L-amino acid counterpart (Jia et al., 2017; Kumar et al., 2018). Furthermore, D-enantiomers of short synthetic AMPs, D-JK-5 and D-JK-6, exhibited increased proteolytic stability and retained anti-biofilm activity against a range of pathogens. However, it’s important to note that D-amino acid substitutions can also impact other AMP properties. Partial substitutions may disrupt α-helicity, while complete substitution creates an enantiomer with potentially different properties (Ting et al., 2020a). For instance, in a study by Hodges et al., D-amino acid substitutions in the AMP V681 led to decreased helical content and increased hemolytic activity, although the reasons for the latter remained unclear (Mant et al., 2019). This approach allows researchers to investigate the impact of these modifications on peptide stability, activity, and structure.

### 4.2 Cyclization

**Figure 8:**
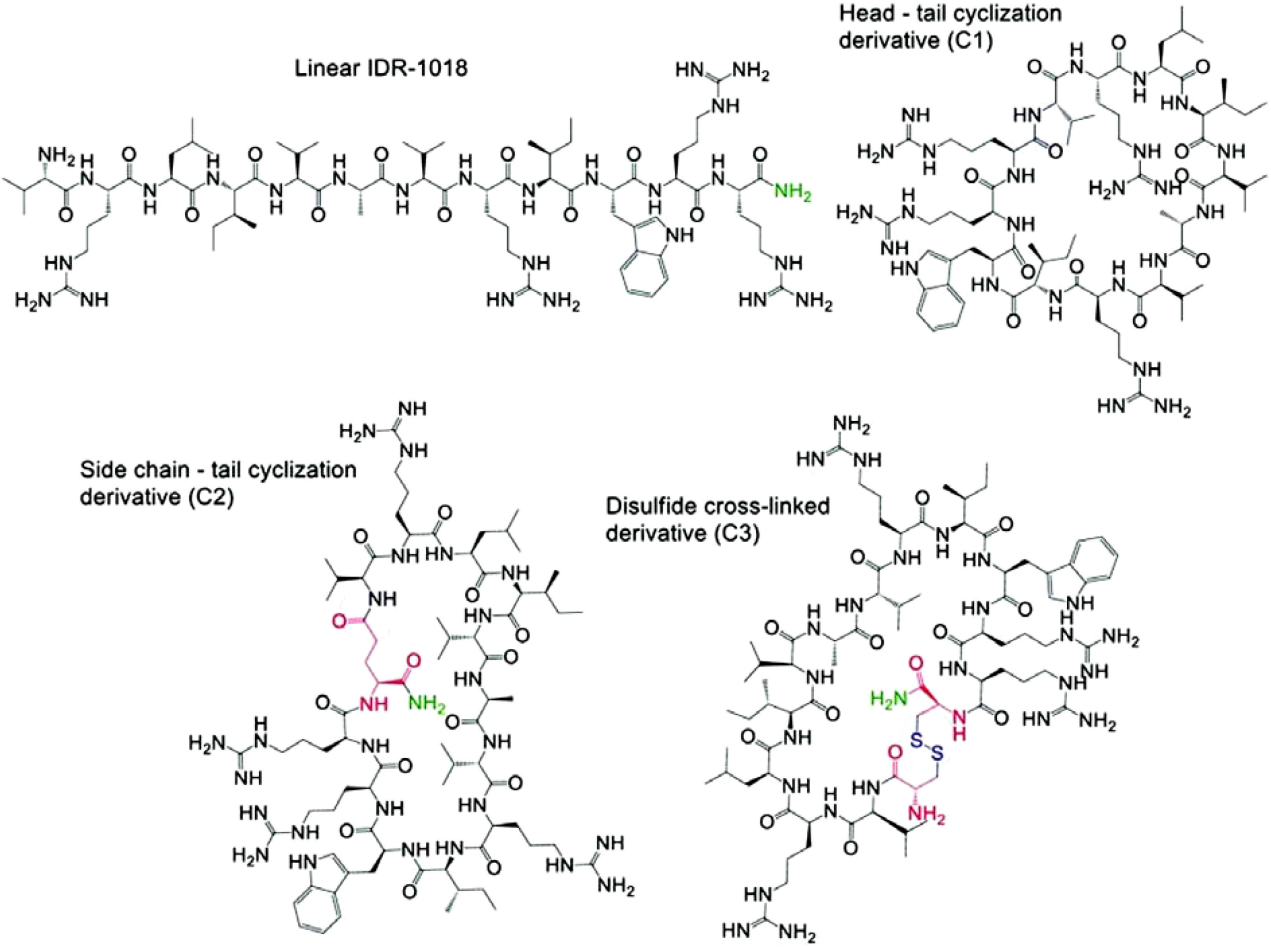
Structural derivatives of linear IDR-1018 peptide. The figure shows various cyclic and cross-linked derivatives of the linear IDR-1018 peptide. (Top) The *Linear IDR-1018* structure is shown as the starting point. (Right) The *Head-tail cyclization derivative (C1)* forms a cyclic structure by linking the N- and C-terminal ends. (Bottom Left) The *Side chain-tail cyclization derivative (C2)* forms a cyclic structure by connecting the side chain with the peptide tail. (Bottom Right) The *Disulfide cross-linked derivative (C3)* features a disulfide bond that connects two side chains, stabilizing the structure. These modifications aim to enhance the peptide's stability and bioactivity.

Macrocyclic peptides show promise as AMPs due to their enhanced stability, reduced flexibility, and improved antibacterial activity and selectivity (Vinogradov et al., 2019; Ting et al., 2020a). This is exemplified by naturally occurring cyclic AMPs like human β-defensins (hBDs), which exhibit these beneficial properties (Fernandez-Reyes et al., 2010; Nyembe et al., 2023). Furthermore, several FDA-approved systemically bioactive AMP medications, including tyrothricin, bacitracin, polymyxin B and E, daptomycin, and gramicidin S, are also cyclic peptides, highlighting the clinical relevance of this peptide architecture (Wang, 2012; Kurpet and Chwatko, 2022; Garcia Jimenez et al., 2023). Moreover, over the past five years, the FDA has granted approval to 17 macrocyclic drugs, further emphasizing their crucial contribution to modern therapeutics (Du et al., 2025). Due to their synthetic accessibility, macrocyclic peptides, are increasingly studied for their antimicrobial potential. Both the peptide sequence and the method of cyclization play crucial roles in optimizing antimicrobial activity while minimizing human toxicity. The potential of macrocyclic drugs for a variety of clinical applications has been emphasized in recent reviews (Garcia Jimenez et al., 2023). This section examines various chemical strategies employed to cyclize AMPs, aiming to enhance their antimicrobial activity and stability while minimizing hemolytic activity. Ghadiri and colleagues designed small, cyclic peptides made of alternating units of L-Trp and D-Leu. These peptides spontaneously form tube-like structures within cell membranes and show potent activity against both Gram-positive MRSA and Gram-negative *E. coli* bacteria. However, these cyclic peptides also exhibited hemolytic activity (Lai et al., 2022). Head-to-tail cyclization has been investigated to enhance the antimicrobial activity of peptides. Cyclized Arg/Trp-rich peptides exhibited significantly greater potency against *B. subtilis* and *E. coli* compared to their linear counterparts, albeit with increased hemolysis (Gan et al., 2021). Similarly, a head-to-tail cyclized peptide composed of alternating hydrophilic and hydrophobic residues exhibited greater activity against MRSA than its linear form (Mohammed et al., 2022). Researchers developed a cyclic AMPs by introducing two cysteine residues into cathelicidin-BF15-a3, a peptide derived from snake venom. This modification, enabled formation of a disulfide bond, yielding a highly potent peptide effective against *P. aeruginosa* and *A. baumannii*, with low toxicity to red blood cells, high stability in living organisms, and a reduced likelihood of inducing bacterial resistance (Mwangi et al., 2019). Hancock and colleagues examined the impact of different cyclization methods, including head-to-tail, side chain-to-tail, and disulfide bond formation, on the properties of the AMPs IDR-1018 (**Figure 8**). Notably, the side chain-to-tail cyclized variant demonstrated potent anti-inflammatory activity and effectively reduced bacterial burden in a mouse skin infection model (Eckert et al., 2006a, 2006b; De La Fuente-Núñez et al., 2014; Etayash et al., 2020).

### 4.3 Terminal/side chain modifications

**Figure 9:**
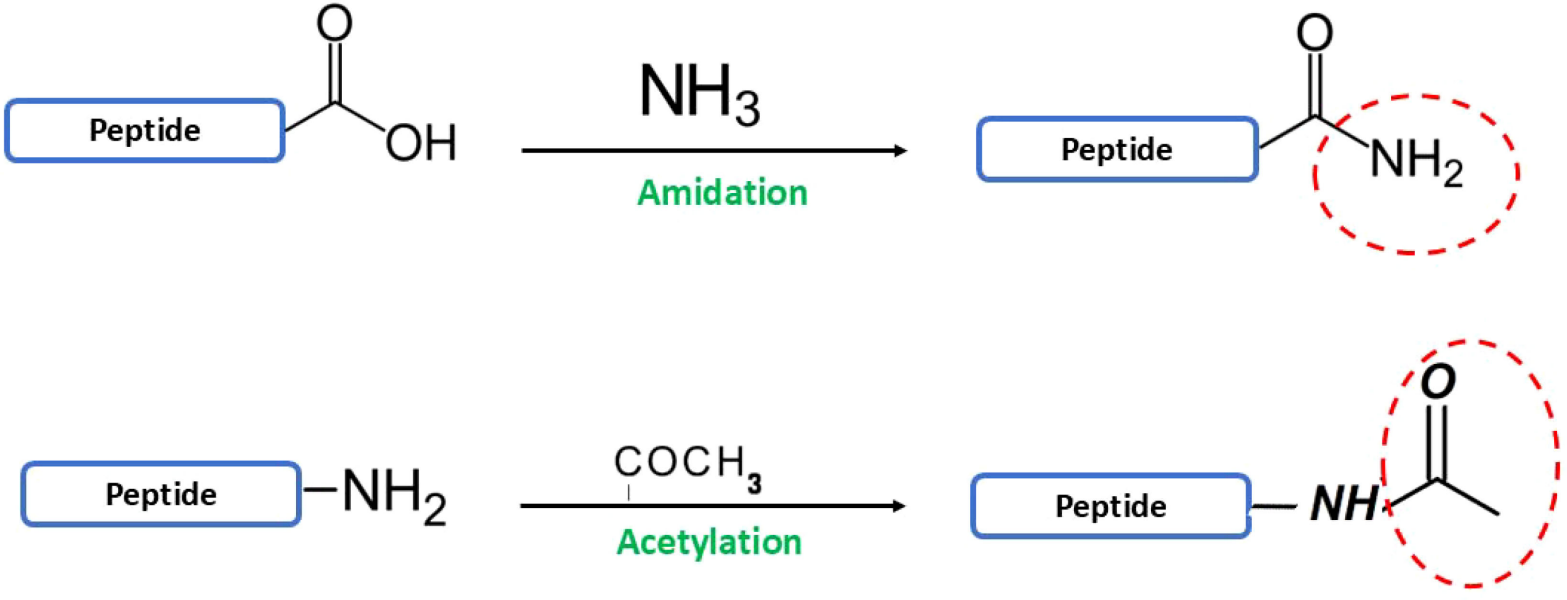
Peptide modification strategies: Amidation and Acetylation. The figure illustrates two common post-synthetic peptide modifications: (Top) *Amidation*, where the C-terminal hydroxyl group (-OH) of a peptide is converted into an amide group (-NH2) through reaction with ammonia (NH3). (Bottom) *Acetylation*, where the N-terminal amine group (-NH2) of a peptide is modified by the addition of an acetyl group (-COCH3), enhancing peptide stability and functionality. These modifications are crucial for optimizing the activity and stability of peptides.

N-terminal acetylation and C-terminal amidation (**Figure 9**) are frequently employed strategies to enhance peptide stability by mitigating proteolytic degradation (Ting et al., 2020a). N-terminal acetylation, a ubiquitous modification in both eukaryotic and prokaryotic cells, effectively inhibits aminopeptidase activity, thereby increasing peptide half-life (Parks and Escalante-Semerena, 2022). However, this modification in concomitantly reduces the net positive charge of the peptide, which may lead to diminished antimicrobial potency (Crusca et al., 2011; Nishita et al., 2017; Baeriswyl et al., 2019). A recent study found that AMPs derived from the MreB protein, a key component of the bacterial cytoskeleton, showed promising activity against a range of microbes. Specifically, the peptide fragment MreB_1-9_, with its +4 net charge, effectively targeted both Gram-positive and Gram-negative bacteria, as well as the fungal pathogen *C. albicans*. However, N-terminal acetylation while preserving antifungal activity, reduced its effectiveness against certain bacterial species like *P. aeruginosa* and *S. aureus* (Nishita et al., 2017). C-terminal amidation, a common modification in natural AMPs, influences their structure and function (Wang, 2012). While N-terminal acetylation can have mixed effects, C-terminal amidation generally enhances the antimicrobial activity of membrane-disrupting peptides. This is likely because it stabilizes their helical structure at the membrane interface, leading to more effective membrane disruption and pore formation (Dennison et al., 2009; Dennison and Phoenix, 2011; Irudayam and Berkowitz, 2012). For example, studies on aurein peptides demonstrate that C-terminal amidation enhances their antimicrobial activity, likely by promoting a stable alpha-helical structure that improves membrane disruption (Sarah et al., 2012; Mura et al., 2016). N-terminal acetylation and C-terminal amidation can have varying effects on antimicrobial activity. However, combining these modifications significantly enhances the stability of AMPs against enzymatic degradation. For example, this dual modification increased the stability of a human apolipoprotein B-derived AMP by more than fourfold in the presence of serum proteins (Oliva et al., 2018). Similarly, modified tachyplesin I showed greater stability than its unmodified form (Kuzmin et al., 2017; Oliva et al., 2018). Additionally, glycosylation and PEGylation of amino acid side chain are employed to increase the ability of peptides to be absorbed and tolerated by the body (Lalonde and Durocher, 2017; Li et al., 2021a). These modifications also reduce immune response likelihood, reduce the rate at which peptides are eliminated by the kidneys, and prolong the time that peptides remain in circulation when administered intravenously (Hagedorn et al., 2010). The utilization of glycoengineering technology facilitates the customization of glycan alterations, hence improving specificity, stability, and antibacterial activity (Correa et al., 2020; Lepeltier et al., 2020; Hadjicharalambous et al., 2022). Moreover, the bioavailability of peptides can be increased by conjugation with large stable proteins. For example, combining the peptide infestin-4 with human albumin extends its half-life from 20 minutes to 276 minutes, significantly prolonging its circulation in the blood (Chen and Jiang, 2023).

### 4.4 Nanoparticle formulations

Recently, the use of nanoparticles (NPs) conjugated with AMPs has gained tremendous attention. Conjugating AMPs to NPs is a highly effective technique for improving the stability of these peptides in host cells, reducing their toxicity, and enhancing their antimicrobial activity. This approach also serves as a promising strategy for targeted drug administration, making AMP-NP conjugates good platforms for delivering drugs to specific targets (Moradi et al., 2016; Sánchez-López et al., 2020; Teixeira et al., 2020a). Furthermore, they do not require transfection as they are internalized within the cytoplasm via endocytosis and are independent of efflux pumps (Le et al., 2017). One of the distinctive physicochemical properties of nanomaterials is their large surface area which helps in the adsorption of more and more AMPs and also prevents their aggregation (Ji et al., 2021). In addition to their inherent antibacterial properties, nanoparticles (NPs) such as silver, zinc oxide, and titanium dioxide have been extensively studied for their effectiveness against a broad spectrum of microbes, including antibiotic-resistant strains. For instance, silver nanoparticles (AgNPs) have demonstrated significant antimicrobial activity by interacting with bacterial cell membranes, leading to cell damage and death. A notable example of an AMPs (AMP) conjugated with silver nanoparticles (AgNPs) causing bacterial cell death involves the AMP indolicidin. Researchers have conjugated indolicidin to AgNPs, resulting in a nanocomposite that exhibits enhanced antibacterial activity against pathogens such as *Escherichia coli* and *Staphylococcus aureus*. The conjugation enhances the peptide’s stability and facilitates its interaction with bacterial membranes. Upon contact, the indolicidin-AgNP complex disrupts the integrity of the bacterial cell membrane, leading to leakage of cellular contents and ultimately causing cell death. This synergistic effect leverages both the membrane-disrupting properties of indolicidin and the inherent antimicrobial characteristics of AgNPs, offering a potent strategy to combat bacterial infection (Pal et al., 2019). Due to their small size, large surface area, and strong targeting capabilities, AMPs encapsulated within these nanostructures are in high demand for developing advanced antimicrobial therapies. The combination of AMPs with NPs not only enhances antimicrobial efficacy but also offers a promising strategy to overcome the limitations of traditional antibiotics.

Additionally, NPs, such as silver, zinc oxide, and titanium dioxide, possess inherent antibacterial properties, which is advantageous to the conjugated AMP system. Moreover, a substantial amount of recently published work has shown the use of silicon-derived nano systems and silver and gold NPs for efficient AMP delivery (Besinis et al., 2014; Shameli et al., 2024). Because of their small size, large surface area, and strong targeting capability, AMPs encapsulated with nanostructures seem to have a great demand in the current context.

### 4.5 post-translational modifications

Several studies have been conducted in this field and it highlights the significance of post-translational modifications (PTMs) in enhancing the productivity and anti-microbial activities of the AMP. Moreover, AMPs require metal ions or PTMs to adopt proper amphiphilic structures, which are mandatory for their antimicrobial activity (Gan et al., 2021). Glycan structure plays a crucial role in promoting the dimerization of glycopeptides in solution. This dimerization effectively increases the valency of the glycopeptide, thereby amplifying its antimicrobial potency (Behren and Westerlind, 2019). One such example includes an antiretroviral drug, enfuvirtide, that is shown to be glycosylated with sialic acid residues, and it has demonstrated improved half-life without affecting the sensitivity and effectiveness towards its target.

Further findings indicated that glycosylation in *P. pastoris*, contributed to the enhanced antibacterial efficacy of the AMP products when compared to those produced in *E. coli.* In addition, *P. pastoris* has shown superiority over both *E. coli* cells and *S. cerevisiae* in various settings. The purified recombinant CHP demonstrated antimicrobial action against both Gram-positive and Gram-negative bacteria, as well as against certain fungi. Both *E. coli* and *P. pastoris* were seen to produce PaDef at elevated levels. However, only the products generated by *P. pastoris* demonstrated notable antibacterial action (Karbalaei et al., 2020a). Furthermore, it was observed that *P. pastoris* and *S. cerevisiae* both successfully expressed EnterocinL50A and EnterocinL50B. However, studies have shown that the bioactivities of the AMPs (AMP) products obtained from *P. pastoris* were 6 and 60 times higher, respectively, compared to those from *S. cerevisiae*. *(* Basanta et al., 2010 *)*. However, glycosylation does not always boost the antimicrobial activity of AMPs because the glycans attached may reduce the hydrophobicity and hence altogether positive charge of the peptides, which has an influence on their interactions and integration into the anionic membranes of the host (Salam et al., 2023). A deeper exploration of how glycosylation affects the function of AMPs is crucial for advancing this field of study.

### 4.6 AI and machine learning for AMP discovery and optimization

The convergence of increased computing power, abundant data, accessible neural networks, and advanced AI algorithms has propelled artificial intelligence to prominence in science and medicine (Ting et al., 2020a; Wang, 2012; Pasrija et al., 2022). Machine learning, in particular, offers a powerful approach for optimizing AMP sequences by leveraging extensive training data (Lin et al., 2020b; Pasrija et al., 2022; Pérez De Lastra et al., 2024). Various machine learning algorithms, including SVM, fuzzy KNN, RF, and NN, have been developed for this purpose (Lin et al., 2020a; Kamran et al., 2022; Xu et al., 2023). For a comprehensive overview of these methods in the context of AMPs, readers are directed to recent reviews (Wu et al., 2019; Jukič and Bren, 2022; Santos-Júnior et al., 2024).

In AMP research, the Quantitative Structure–Activity Relationship (QSAR) model is frequently employed to predict peptide activity by analyzing their chemical structures and molecular features (Wu et al., 2019; Keyvanpour and Shirzad, 2021; Jukič and Bren, 2022). QSAR modelling has been successfully employed to optimize AMP properties (Taboureau, 2010). For instance, Ding et al. demonstrated that substituting hydrophobic amino acids with less lipophilic residues can reduce hemolytic activity without compromising antimicrobial activity (Biasizzo and Kopitar-Jerala, 2020). Applied a QSAR model to uncover peptide motifs capable of forming silver nanoparticles, broadening its use beyond antimicrobial functions. Furthermore virtual screening using QSAR modelling has emerged as a powerful approach for discovering and optimizing AMPs (Vincenzi et al., 2024a). For example, a computational approach utilizing a QSAR model to screen a virtual library of peptide structures successfully identified promising candidates with enhanced antibiofilm activity. This highly accurate model (85% success rate) led to the discovery of peptide 3002, which demonstrated an eightfold increase in eradicating established MRSA biofilms *in vitro* compared to its parent peptide (Haney et al., 2017; Mansour, 2018). Using Mutator, a web-based computational tool for optimizing AMPs selectivity, Tossi and colleagues identified Dadapin-1 (Waghu et al., 2020). While computer-aided design shows promise for developing enhanced antibiofilm peptides, further refinement of predictive models is anticipated with the increasing availability of active peptide sequences in databases (Reymond et al., 2010).

Virtual screening of vast molecular libraries within chemical space accelerates the discovery of novel AMPs, identifying promising candidates for synthesis and testing (Reymond et al., 2010; Kale et al., 2023). One successful example is the work of the Reymond group, who have utilized this approach to discover and optimize bicyclic peptides—a class of molecules with high stability due to their constrained structure. Their research led to the identification of compounds 27b and 62b, which exhibited potent activity against multi-drug resistant *P. aeruginosa* and other clinically relevant Gram-negative bacteria (Di Bonaventura et al., 2017). In a separate study, they screened a virtual library of over 4.6 million bicyclic peptides, leading to the identification of bp56, a potent and stable compound active against MDR strains of *A. baumannii* and *P. aeruginosa* (Di Bonaventura et al., 2018). The Reymond group also employed a similar approach to enhance the activity of the AMPs dendrimer G3KL. This resulted in the discovery of T7, a dendrimer with significantly improved efficacy against a panel of Gram-negative bacteria, including MDR *P. aeruginosa* and *K. pneumoniae* strains (Siriwardena et al., 2018).

## 5 Conclusion

AMPs hold significant promise as a novel solution to the growing global threat of antibiotic resistance. Their broad-spectrum activity against bacteria, fungi, and viruses, coupled with their unique mechanisms of action, positions them as a valuable alternative to conventional antibiotics. While challenges remain in production and optimization, advancements in genetic engineering, chemical synthesis, and rational design are paving the way for AMPs to become viable therapeutic agents. Overcoming toxicity and stability hurdles through innovative research strategies, including structural modifications and targeted delivery systems, is crucial for realizing the full clinical potential of AMPs. Continued interdisciplinary efforts in microbiology, medicinal chemistry, and synthetic biology will be essential to refine AMP design and unlock their potential to combat drug-resistant infections effectively. The future of AMPs as a powerful tool in human healthcare hinges on continued research and development, ultimately offering hope for a new era of effective antimicrobial therapies.
